# The experience of loneliness among people with a “personality disorder” diagnosis or traits: a qualitative meta-synthesis

**DOI:** 10.1186/s12888-022-03767-9

**Published:** 2022-02-17

**Authors:** Sarah Ikhtabi, Alexandra Pitman, Gigi Toh, Mary Birken, Eiluned Pearce, Sonia Johnson

**Affiliations:** 1 UCL Division of Psychiatry, London, UK; 2 Camden and Islington NHS Foundation Trust, London, UK

**Keywords:** Loneliness, Personality disorder, Qualitative synthesis, meta-synthesis, Thematic synthesis

## Abstract

**Background:**

Loneliness is prevalent among people with a “personality disorder” diagnosis or who have related personality traits, but the experience of loneliness among people with “personality disorder” diagnoses/traits has not been well described. A qualitative approach has potential to help understand the experience of loneliness among people with “personality disorder” diagnoses/traits, and to develop interventions that promote recovery. We therefore aimed to synthesise the qualitative literature relevant to this topic.

**Method:**

We conducted a meta-synthesis of qualitative studies exploring the subjective experience of loneliness as reported by people with “personality disorder” diagnoses/traits. We searched four databases using pre-formulated search terms, selected eligible articles, appraised the quality of each, and analyzed data from eligible studies using thematic synthesis.

**Result:**

We identified 39 articles that described the experience of loneliness in people with “personality disorder” diagnoses/traits. From extracted data, we identified seven themes: (1) disconnection and emptiness: a “haunting alienation”, (2) alienation arising from childhood experiences, (3) thwarted desire for closeness and connection, (4) paradox: for both closeness and distance, (5) experiences of existential loneliness, (6) recovery, embedded in a social world, and (7) group therapy: a setback. Our results suggest that for our sample early alienating and traumatic experiences may pave the way for experiences of loneliness, which further exacerbate “personality disorder” symptoms and distress.

**Conclusion:**

Despite describing a need to belong and efforts to cope with unmet social needs, people with “personality disorder” diagnoses/traits (particularly “emotionally unstable personality disorder”) report experiencing an intense disconnection from other people. This seems rooted in early adversities, reinforced by later traumatic experiences. Given the apparent salience of loneliness to people with “personality disorder” diagnoses/traits, interventions focused on helping people connect with others, which may include both psychological and social components, have potential to be beneficial in reducing loneliness and promoting recovery.

**Supplementary Information:**

The online version contains supplementary material available at 10.1186/s12888-022-03767-9.

## Introduction

Loneliness is a painful, subjective, emotional state characterised by a perceived discrepancy between desired and actual patterns of social interaction [1]. It seems to both increase the risk of, and hamper recovery from, a range of mental health problems [2–4]. Loneliness has been identified as a potential intervention target in mental health conditions including depression, anxiety, dementia, eating disorders, and psychosis, both because it is an inherently distressing state with established associations with poor general health outcomes, and because reducing loneliness is a potential route to preventing or alleviating mental health problems [5–10]. Qualitative explorations of the experience of loneliness in disorders such as depression provide insights into potential pathways linking loneliness and mental health conditions and can suggest potential treatment approaches [11]. However, although an association between loneliness and “personality disorder” has been described [12], we know little about how people with “personality disorder” diagnoses or traits experience loneliness.

A substantial proportion of the population demonstrate traits associated with a “personality disorder” diagnosis. A recent systematic review estimated a pooled prevalence of “personality disorders” in the general population at 8%, and up to 10% in high-income countries [13]. Fewer studies estimate the prevalence of individual traits characteristic of “personality disorder”, but 37% of people in a UK survey that used a validated screening tool reported four or more traits characteristic of “personality disorder”, reaching the threshold for possible diagnosis of “personality disorder” [14]. In general clinical practice, the most frequently encountered conditions, are the Cluster B conditions, such as “emotionally unstable personality disorder (EUPD)” [15], and these are also the focus of much of the research on “personality disorder”. However, there are no clear boundaries between these clusters, and Cluster B conditions, such as “EUPD”, overlap with Cluster A and Cluster C conditions, such as “avoidant personality disorder (AVPD)” [16]. Indeed, a high proportion of individuals diagnosed with one particular “personality disorder” also meet criteria for another “personality disorder” diagnosis and other mental health problems [17, 18].

The usefulness and psychological robustness of a “personality disorder” diagnosis is frequently criticized in both scientific literature and commentaries by people with lived experience [19–22]. Its criticisms include the heavy weight of stigma and hopelessness that has come to be associated with the diagnosis, the lack of acknowledgement of the important role of trauma and adversity, and the lack of progress in delivering good care associated with the use of this diagnosis [21–24]. As such, alternative descriptions such as “complex emotional needs” are advocated and increasingly in use [21]. For this review, we have decided to retain and use the term “personality disorder” in view of the absence of an agreed upon alternative acceptable term up until this point, and the fact that the literature reviewed uses the label “personality disorder”. However, in acknowledgement of the criticisms of this label, our use of quotation marks reflects our view that terminology in this area, and values underpinning this, require review.

Difficulties with interpersonal relationships are frequently reported among people diagnosed with a “personality disorder” [25–27]. People with a diagnosis of “EUPD” have especially been investigated through social network analysis and psychological experimental studies, and are reported to have poorer social support, less social integration, greater dissatisfaction and fear during interpersonal interaction, and higher levels of loneliness compared to people without a “personality disorder” or people with few “EUPD” traits [28–31]. Contemporary social needs approaches suggest that longer-term effects of adverse childhood experiences, and the accompanying development of early maladaptive schemas, often include loneliness and interrelated schemas of rejection and abandonment [32–36]. These schemas echo stable and painful ideas that one is not connected to other people or not worthy of being loved, which underpin unhelpful ways of relating to others [32]. Related research findings relevant to understanding loneliness among people with a “personality disorder” diagnosis, especially “EUPD”, include associations between loneliness or lack of social connectedness and greater rejection sensitivity, adverse emotional reactions to social exclusion and inclusion, and lack of confidence in social interaction and biased evaluation of facial expressions [28, 33, 37–40]. Together this evidence suggests that in people with a “personality disorder” diagnosis, inherent difficulties with developing satisfying social relations and a sense of belonging are linked to feelings of loneliness via a range of cognitive processes, which may interact with the stigma and actual objective experiences of rejection often encountered by people diagnosed with a “personality disorder” [41, 42].

Quality of care for people diagnosed with a “personality disorder” has persistently been criticised as deficient by service users, clinicians, researchers, and policy makers in several countries [21]. One prominent call has been for the help available to have a broader focus, as specialist treatments are criticized as focusing too narrowly on preventing self-harm [21]. A broader range of interventions is particularly important as challenges in living that concern people with relevant lived experience most often include difficulties feeling connected to others and maintaining relationships [21]. Given the prevalence of loneliness among people with “personality disorder” diagnoses and its association with a range of adverse health outcomes, loneliness is a promising potential focus for general intervention [43, 44]. However, as yet, there are few reports on interventions tailored to people with a “personality disorder” diagnosis. The importance of understanding and potentially intervening to reduce loneliness in this context is underlined by findings that loneliness and difficulties in social connectedness is associated with suicidal and self-injurious behaviours among people with an “EUPD” diagnosis [45, 46] and criminality among people with cluster B “personality disorder” diagnoses [47].

To underpin further work aimed at understanding the relationship between loneliness and the cluster of difficulties associated with “personality disorder” diagnoses, it is important to understand the relevant experiences of people with “personality disorder” and their accounts of what helps them to feel less lonely [48]. One review emphasized that more research is needed to investigate constructs such as chronic emptiness, a diagnostic criterion of “borderline personality disorder” in DSM-IV that is characterized by a feeling of internal absence and hollowness [49, 50], and loneliness and whether chronic emptiness encompasses experiences of loneliness [51]. Qualitative work conducted since then suggests that chronic emptiness is distinct from loneliness [50]. Although experiences and emotions associated with chronic emptiness, such as a sense of disconnection from others and deadness, may be similar to difficulties associated to loneliness experiences [50, 51], emptiness is a form of disconnection from both the self, including one’s own values and goals, and others, while loneliness is described as a disconnection from the world and others [50]. However, further work is clearly needed to understand the relationship between these two constructs, particularly in relation to “personality disorder” for which emptiness is a key component.

Two reviews of the qualitative literature exploring the experience of personal recovery in people with a “personality disorder” diagnosis have described a sense of feeling safe as an essential component of recovery and reported that this was most readily achieved in the context of strong social network support [48, 52]. To our knowledge, no literature review has yet synthesized qualitative papers on the experience of loneliness among people with a diagnosis of “personality disorder”, either for the Cluster B presentations which are the focus of most research on “personality disorder”, or for the fuller spectrum of “personality disorders”.

To address this evidence gap, we aimed to conduct a meta-synthesis of the qualitative literature to draw together evidence exploring the subjective experience of loneliness as reported by people with a diagnosis or the associated traits of “personality disorder”. In providing a richer understanding of the impact and role of loneliness in the day-to-day lives of people with a diagnosis or traits of “personality disorder”, our aim is to underpin further work focused on understanding this relationship and on developing and adapting therapeutic approaches tailored for the needs of this group.

## Methods

### Design

We conducted a meta-synthesis of qualitative studies exploring loneliness experiences among people with diagnoses/traits of “personality disorder”. Meta-synthesis is a rigorous research method that entails the analysis and synthesis of findings from qualitative literature to generate new higher-order understanding via the interpretation of qualitative evidence [11, 53–56]. Meta-synthesis is adapted from thematic synthesis, which embraces a combination of characteristics from grounded theory and meta-ethnography [53]. This process requires organization of free codes into descriptive themes, followed by further interpretation of commonalties and patterns to allow for the construction of analytic themes [53, 54]. This is achieved by an iterative, cyclical process that involves careful familiarization, line-by-line coding, grouping and categorizing codes across studies to obtain descriptive themes, and higher-level synthesis to achieve new emerging themes and knowledge [11, 53, 54, 57, 58].

We synthesized qualitative data employing an established six step approach to conducting a meta-synthesis, which offers a balance between objectivity and necessary researcher subjectivity [54]. This process involved: (1) defining a research question and selection criteria, (2) employing the criteria to select studies, (3) conducting a quality assessment, (4) extracting and presenting formal data, (5) conducting data analysis, and (6) expressing the synthesis of findings [55].

### Database and search strategy

We registered our meta-synthesis protocol on open science framework (OSF) (https://osf.io/gknp9/?view_only=ac55c51325854c94aaf701659b5517a2). We conducted the search using the following four electronic bibliographical databases, from inception to 03 June 2020: Medline, Embase, PsychINFO, and Web of Social Science. To reduce the risk of missing any relevant articles, we also searched Google Scholar, Ethos British Library database, and the reference lists of eligible articles, to identify articles, MSc dissertations and PhD theses on this topic.

Search terms were finalized after conducting a preliminary test search, which allowed the team to review terminology in relevant studies and ensure all relevant terms were included. Conceptually overlapping or closely associated terms, including social isolation and personal recovery, were also included in the search to ensure comprehensive retrieval of relevant papers, but only material specifically focused on loneliness was included in the meta-synthesis. Current practices to conceptualising and assessing “personality disorder” include categorial diagnostic approaches and dimensional approaches based on assessing severity of traits. To increase comprehensiveness of our search, we included not only studies of people meeting formal diagnostic criteria for “personality disorder”, but also people scoring above a specified threshold on dimensional assessments of personality traits based on clinician- or self-assessment. Additional file 3: Appendix 1 shows search terms in full, including modifications for specific search engines.

### Selection: inclusion and exclusion criteria

Studies were screened in accordance with the following inclusion criteria:

- At least 50% of participants met diagnostic criteria for any form of “personality disorder”, scored above a specified threshold on dimensional measures of problematic personality traits or self-reported as having “personality disorder” or “personality disorder” related traits, including participants with additional comorbid conditions such as depression, attention deficit hyperactivity disorder (ADHD), or substance abuse.
- The focus in at least part of the paper was on exploring participants’ reported experiences of loneliness, current or retrospective.
- Studies using any recognized qualitative design, including data collection through semi-structured interviews, written data sources, or focus groups, and any recognized method of qualitative analysis, including thematic analysis or interpretative phenomenological analysis.
- Studies were published in English.

We excluded the studies with:

- A specific and sole focus on individuals with “personality disorder” diagnoses/traits within the criminal justice system (including prisons). We expected that this sample would have very distinctive experiences of loneliness shaped by incarceration experiences and coping with experiences associated with incarceration, perceived social estrangement (particularly if serving time in prison), and lack of control over who to interact with [58].
- Studies focusing on individuals with both “personality disorder” and co-morbid chronic physical conditions, developmental disorders (except for ADHD as above) and neurocognitive disorders or traumatic brain injury.

### Data screening

After removing duplicated references identified in the database searches, one researcher (SI) screened the titles and abstracts of all citations identified against the inclusion criteria to identify potentially eligible articles. A second researcher (GT) independently screened titles and abstracts for a random 10% of all citations to check for agreement, discussing any disagreements with SI and the wider review team. SI then conducted a full-text screen of remaining papers to determine a final set of eligible articles meeting the inclusion criteria. Again, GT independently screened a randomly selected 10% of articles to check for agreement, discussing any disagreements with SI. SI then reviewed reference lists for all eligible articles and GT independently reviewed reference lists for a randomly selected 10% of articles. Regular meetings and discussions were held with the wider review team to ensure adherence to criteria and resolve areas of uncertainty.

### Data extraction

A data extraction proforma was used to summarize information and characteristics for each study, including: citation, study setting, study aim(s), sample size and type of diagnosis/self-reported diagnosis/traits, characteristics of the sample, data collection method, data analysis method, major themes identified in the original study, and quality assessment rating as outlined below. A second researcher (GT) independently conducted the data extraction for a randomly selected 10% of eligible articles to check for agreement, discussing any disagreements.

### Quality appraisal

One researcher (SI) conducted a quality appraisal of each eligible article using the Critical Appraisal Skills Programme (CASP) checklist; a widely-used tool to facilitate rapid evaluation of qualitative data to assess study quality [59]. We did not exclude articles on the basis of low quality as the aim of this review was to synthesize comprehensively a range of experiences as reported in all relevant studies. However, we used the CASP tool to improve the rigor of this review by presenting findings in the context of an assessment of each study’s quality [11], in line with methodological guidance for conducting a meta-synthesis [11, 58]. A second researcher (GT) independently appraised the quality of 10% of all eligible articles to check for agreement, discussing any disagreements.

### Data analysis

One researcher (SI) familiarised herself with all the data by carefully reading eligible papers. Any text relevant to the study aims was identified and extracted into a qualitative data analysis software package, NVivo, to facilitate processing of data [60]. A second researcher (GT) independently reviewed a randomly selected 10% of eligible articles to establish which passages of text should be extracted and included in the dataset, resolving any disagreements through discussion. SI then coded included data using NVivo, building up a framework of descriptive themes. These themes were further compared and contrasted to create overarching themes. Emerging descriptive and subsequent analytic themes were discussed with the review team, including discussions to address reflexivity.

### Reflexivity

The multidisciplinary team included those with a variety of viewpoints based on their clinical and research experience in relation to loneliness and “personality disorder”. The wider team consists of academics with experience and interest in the mental health impacts of loneliness, with some members conducting research on “personality disorder”. SI, an Arab female, was an MSc student in Clinical Mental Health Sciences and is interested in the relationship between loneliness and “personality disorder”. She is now a PhD student conducting research in the same area. AP and SJ are white female psychiatrists with clinical and research experience relevant to the study topic, MB is a white female with a background in occupational therapy and with clinical and research experience relevant to the study topic, EP is a white female anthropologist with research expertise in loneliness, and GT is a Malaysian Chinese female who that has conducted research on the effectiveness of digital interventions for social isolation. Team members from White backgrounds have a variety of European origins. All team members would be classified as middle class, or similar, by occupation-based classifications. This helped gain a richer understanding of material and mitigated the high risk of subjectivity inherent to a meta-synthesis [54]. It is important to note that from the outset, we acknowledged concerns regarding the pejorative nature of the term “personality disorder”, but in aiming to synthesize evidence from previous qualitative studies, we employed language and labels used originally by the authors in the included studies.

## Results

### Study characteristics

**Fig. 1:**
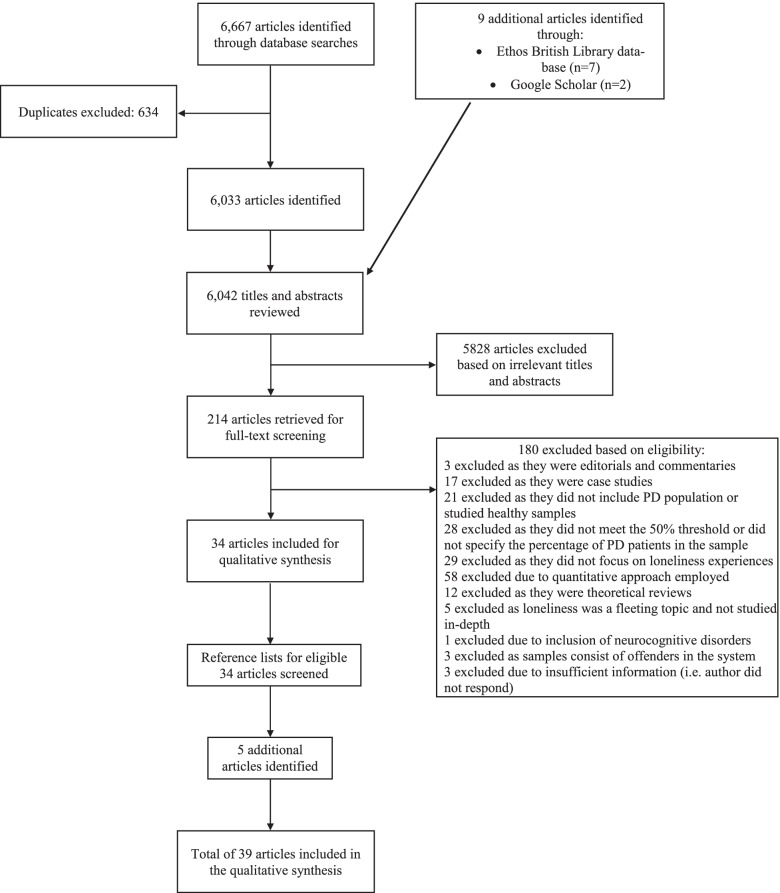
Flow Diagram of studies through systematic review process

We identified a total of 6042 articles from the four databases (*n* = 6033) and the additional sources (*n = 9).* After screening titles and abstracts, we derived a total of 214 potentially eligible articles, and conducted a full text screen of these, identifying 34 articles as meeting inclusion criteria. We achieved more than 95% agreement on decisions to include/exclude articles to ascertain eligibility in the title and abstract screening process; and through discussions achieved 100% agreement on decisions to include/exclude eligible articles. The study selection process is summarized in Fig. 1. Review of reference lists yielded an additional 5 eligible articles, resulting in a total of 39 included articles.

Characteristics and CASP scores for each study are described in Table S1 (Additional File.1). Studies generally had clear aims and findings and used appropriate qualitative methodology, but many scored poorly in considering issues of reflexivity. The total confirmed number of participants in included studies was 721, with sample sizes ranging from 3 to 100. However, three studies did not specify their sample size [36, 61, 62]. The majority (*n* = 24) of studies took place in the United Kingdom (UK). The majority exclusively (n = 24) focused on samples with a diagnosis or traits related to “EUPD”. However, five studies did not specify whether participants were clinically diagnosed with or self-reported a “personality disorder” [63–67]. One study included a sample of young people at risk of a “personality disorder” [65]. Only three studies specified using a dimensional measurement to assess “personality disorder” related traits [68–70]. Two studies included general population participants as control groups [71, 72] and one study included therapists and relatives’ perceptions [70]. Most studies used interviews or semi-structured interviews (*n* = 34) to collect data. A large selection of qualitative analytic approaches was used, with the majority of studies using thematic analysis or interpretative phenomenological analysis.

### Thematic framework

Through the approach of thematic synthesis, we identified seven themes, described below along with illustrative quotes from included papers. Table S2 (Additional File. 2) presents these themes together with their sub-themes and further illustrative quotes from authors and participants. Direct quotes from the research study authors’ interpretations of data are identified using speech marks only and direct quotes from research participants are given in italicised text and speech marks (followed by identifiers where available).

### Theme 1: disconnection and emptiness: a “haunting alienation”

Most studies conveyed a strong sense of participants’ “lonesome struggle”, characterized by feeling persistently estranged and disconnected [73]. Whether or not they were objectively lacking in contact with others, participants recurrently expressed an intense feeling of otherness, characterised as a *“haunting alienation”* in which a person did not feel like a “*part of anything*” [36]. This brought “about a sense of disconnection”, emptiness and “dissociation from society at large” [36, 69, 74]. These experiences were especially described by people with a diagnosis/traits related to “EUPD”:

*“Constant loneliness; even in a crowded room; always – I’ve always felt completely detached from everybody – everything and everybody [ … ] there’s always that part of me that doesn’t feel I belong and that makes me feel lonely”* (interviewee from a UK sample with a diagnosis of “EUPD”) [36]

Participants from Norwegian [73] and UK samples [36, 42, 70] with “personality disorder”, particularly “AVPD” and “EUPD”, spoke of this experience of loneliness and dissociation as a chronic and inherent part of who they were, almost as if it were something inevitable and deserved. To demonstrate, one participant described that loneliness is:

*“what my life is, what’s, what I was destined for. You know, this is almost my punishment for surviving the rest of it.”* (interviewee from a UK sample with a diagnosis of “EUPD”) [36]

Participants from an American sample with “EUPD” and a Canadian sample with Cluster B “personality disorder” described feelings of inadequacy that seemed to create a gap separating them from others [66, 75]. Specifically, they described “falling short” in comparison to others which created a sense of disconnectedness.

*“I just rate them against me and I have never met anyone that I was equal to or better than, no matter what … even if it was a bum on the street”* (interviewee from an American sample with a diagnosis of “EUPD”) [75]

In an Indian [71], Norwegian [73], Swedish [76], American [75] and four UK samples [36, 42, 77, 78] participants with a “personality disorder” diagnosis often described disconnection and loneliness experiences in association with feeling misunderstood by everyone, invisible, and a burden on others. People with a diagnosis of “AVPD” and “EUPD” particularly expressed that they felt as if they were not known or seen by others.

*“I can be walking down yet I can see all these activities going on but it’s like I’m not there and nobody can see me. I get very lonely.”* (interviewee from a UK sample with a diagnosis of “EUPD”) [42]

In narratives of Australian and Norwegian samples of participants with an “EUPD” and “AVPD” diagnosis, respectively, some individuals associated these experiences with a sense of already being dead [73, 74]. Moreover, a participant from a UK sample with “EUPD” described engaging in self-harm as a way to cope with intense disconnection and subsequent emptiness and the sense of deadness, all of which are key element of emptiness [36, 42]:

*“I just felt different from everybody else [ … ] it was just that I had an incredible empty space inside me that didn’t seem to be filled with anything that I did. I began to feel more and more different and more lonely [ … ] I used to self-harm because that made me feel real in that moment.”* [36]

Here, the sense of emptiness seems to mark the individual out as different, which made them feel lonely and disconnected.

### Theme 2: alienation arising from childhood experiences

The data coded under this theme illustrated that the sense of disconnection and separateness was often described as originating from alienating childhood experiences that seemed to contribute to feelings of uncertainty regarding a person’s sense of their place within a family or social unit. Participants from UK samples with “personality disorder” and “EUPD” [64, 79–81], an Australian [74] and a Norwegian sample of women with “EUPD” [63] expressed a sense of exclusion that seemed to arise from early life experiences with their families; this eventually led to a sense of being *“totally different”*. Participants described a “pervasive and chronic form of rejection, in which they felt excluded from sibling relationships, and from their parents’ marital dyad and they also felt scapegoated by entire family systems and sub-systems” [66]. Participants from other UK studies [64, 79] also expressed similar narratives in which they felt scapegoated within their family unit.

*“I feel like the black sheep of the family to be honest”* (interviewee from a UK sample with a diagnosis of “personality disorder”) [79]

Participants from UK studies [36, 42, 70, 78, 81, 82], including a sample of youth at risk of a diagnosis of “personality disorder” [83], an Indian sample with “personality disorder” [71], an Australian sample with “EUPD” [74], and a Norwegian sample with “AVPD” [73] described early traumatic experiences along with psychological and emotional separation from the family unit, and related these to their feelings of loneliness. A number of participants, particularly those diagnosed with “EUPD”, recalled these experiences as being characterised by emotional loneliness or an absence of intimate relations [36, 74, 78, 81, 82, 84]. For instance, some emphasized having *“no family”*, feeling *“very unwanted”*, neglected and misunderstood by the family, and *“having nothing in common with the family”* [36, 42, 61, 64, 70, 71, 73, 74, 79, 80, 83]. Emotional loneliness that originated from childhood experiences and absence of caring adult relationships were sometimes described alongside experiences of childhood neglect and traumatic childhood physical and sexual abuse:

*“When I got molested as a child, I could never speak to them* (parents) *because I felt they would not understand. I grew up with a “yuck” feeling about myself that did not allow me to form deep bonds with anyone.”* (interviewee from an Indian sample with a diagnosis of “personality disorder”) [71]

### Theme 3: a thwarted desire for closeness and connection

People with a diagnosis/traits of “personality disorder” expressed a strong but thwarted desire for social connections and intimate relationships [64, 67, 68, 71, 73, 74, 76, 85]. This longing for fellowship, apparent particularly in participants diagnosed with “AVPD” and “EUPD”, seemed to be accompanied by a struggle to make desired connections successfully [73, 85]:

*“I would like to interact better with people, be more forthcoming, more sociable, more gregarious, less paranoid.”* (interviewee from an Indian sample with a diagnosis of “AVPD”) [71]

*“(I’d like to) maybe establish more interpersonal relationships, real friendships with people, and maybe a romantic relationship would be nice”* (interviewee from a UK sample with a diagnosis or self-reported “personality disorder”) [67]

Specifically, participants described that they yearned for romantic relationships and friendships rooted in mutual understanding [67]. Despite this desire to connect and engage with others on a deeper level, participants revealed that they did not know how to invest in relationships or found it hard to understand others’ intentions [64, 71, 73]. In a Norwegian sample of people diagnosed with “AVPD”, “some expressed that they were fond of people and wished to be liked”, yet “most felt upset by how they could not manage to socialize” [73]*.* One participant from an Indian sample with a diagnosis of “personality disorder” reflected that this inability to socialize might be “*because my parents never taught me how to understand other people.”* [71].

### Theme 4: paradox: pull for both closeness and distance

This theme reflected a paradoxical combination of a yearning for closeness and connection, conflicting with a strong fear of intimacy [36, 63, 73, 74, 76, 78, 86, 87]. This simultaneous conflicting pull for closeness and distance was very challenging for participants to resolve. This was illustrated by one participant who shared that *“it’s just that emptiness and … … it’s almost the desperation of wanting to allow people in but not being able to. I just think I’ve been lonely all my life”* (interviewee from a UK sample with a diagnosis of “EUPD”) [36].

The need to be distant seemed eventually to cause feelings of inner emptiness and loneliness, which then triggered the desperate need to connect with others in order to counter for these painful feelings [36, 73, 86, 88]. However, this desperation for connecting was resisted due to feelings of terror and anxiety [63, 73, 76, 78, 86, 87]. This paradox was described in association with core symptoms of “personality disorder”, such as fear of rejection, abandonment, and “others’ possible opinions and motives”, suggesting that these core vulnerabilities might underpin this paradox [63, 67, 73–75].

In the subthemes below, we discuss the specific fears and perceptions that are reported to contribute to this fear of intimacy demonstrated by people with a diagnosis/traits of “personality disorder” as well as the ways in which they attempted to cope with this relational paradox.

## Subtheme 4.1: a rejecting and hostile external world

Participants with a “personality disorder” diagnosis/traits perceived the relational world as hostile and rejecting, and described adverse experiences with others in both childhood and adulthood. This seemed to contribute to an urge to withdraw to maintain a sense of safety from others [36, 66, 67, 69, 73, 80, 81, 87]. In keeping with the perception of a hostile world, participants from a Norwegian sample with “AVPD” [73], Australian sample of women diagnosed with “EUPD” [74], an American sample [75], a UK [67], and Dutch sample [89] with “EUPD”, experienced threat-related physical reactions and intense fear “building up as the moment of some interaction” drew near. Even when participants were well aware that a social situation was not actually dangerous, “their bodies gave the opposite message of imminent threat” [36, 66, 67, 73, 80, 87]:

*“Every time I leave a conversation or something, I go out to breathe and tell myself, “It is not dangerous; it is not dangerous.” Then, I calm myself, and it gets just as bad again. I get very tense and I sweat, like it is dangerous.”* (interviewee from a Norwegian sample with a diagnosis of “AVPD”) [73]

The perception of a hostile and rejecting world was described as being reinforced by adverse experiences with unsupportive and dismissive caregivers and emotionally rejecting experiences with partners and families, both in childhood and adulthood [61, 63, 65, 70, 82, 83, 89]. These experiences often motivated participants to “push away relationships through fear of rejection” [69]. Indeed, “many clients reported feelings of isolation and loneliness”, often due to past and current difficulties in personal relationships [61]. This was apparent in the observations of a research participant who took notes on a therapeutic community meeting: “Tessa explains she felt unwanted and unloved by people here and by her family …. She could hear people laughing outside her door and she felt excluded” [61].

Participants mentioned that stigmatising, negative attitudes and lack of sensitivity from professionals further increased fears of rejection and abandonment [63, 84]. Professionals sometimes seemed to be re-enacting rejection behaviors that resembled or triggered memories of abandonment. A participant from a Norwegian sample of women with “EUPD” demonstrated how past experiences of rejection seeped into her day-to-day life, challenging the way she related to others and increasing her distress to the point of self-harm:

*“I thought she would never return again and I trembled with fear, wondering where she was going, feeling abandoned and alone. The only thing left was to cut myself to obtain relief”* (interviewee from a Norwegian sample of women “suffering” with “EUPD”) [63]

As well as coping with repeated abandonment, participants perceived the relational external world as demanding [36, 65, 80, 82], reporting a feeling that they had to please others, rehearse for social conversations and behaviours, and deal with intrusive people [73–75, 82].

*“You just spend your entire consciousness in just not … trying not to make a fool out of yourself and appear normal”* (interviewee from a Norwegian sample with a diagnosis of “ AVPD”) [73]

### Subtheme 4.2: ways of managing the paradox

This sub-theme described how people with a diagnosis/traits of “personality disorder” actively employed strategies to cope with their urge to connect and counter loneliness whilst simultaneously keeping their distance [66, 68, 75, 86]. These coping methods, described primarily by people with a diagnosis of “EUPD” and “AVPD”, were perceived as safer ways of connecting and were often motivated by a need for self-preservation and protection from potential future trauma resulting from forming intimate connections [36, 67, 73, 74, 76, 81, 82, 86]. In two UK studies [68, 70], a Norwegian [73], and an Australian study [74], participants with a diagnosis/traits of “EUPD”, “AVPD”, and other “personality disorder” seemed to compensate for difficulties with human connection by forming connections with pets. This narrative by a participant from a UK sample with traits of “EUPD” is reflective of the essential need to avoid feeling alone:

*“it’s a bit depressing [ … ] I ain’t got a girlfriend or anything. I hadn’t for a while, so, do you know what I mean? Makes you a bit lonely, but that’s why – you know what I mean? I compensate. And I have four dogs”* [75]

In a Canadian sample of people with Cluster B “personality disorder” some preferred interacting through a virtual life using digital technology [66, 73]:

*“I try to talk about it as little as possible: my cell phone is something that takes up a lot of space in my life. I have a virtual life as one might say”* [66]

Participants from UK samples [36, 64], a Norwegian [73], and an American sample [75], primarily with a diagnosis/traits of “EUPD” and “AVPD”, preferred forms of social connections that were “safe” and structured, such as volunteer work and arts activities, as it allowed them to satisfy their urge to connect. However, where intimacy naturally grew through these activities, the fears could still arise:

*“but after like half a year, everyone had sort of made their small groups of friends already, and then, it seemed a bit strange that I did not have that, so then it was better for me to pretend and lie”* (interviewee from a Norwegian sample with a diagnosis of “AVPD”) [73]

Sometimes participants would deliberately set unhelpful limits on their relationships [69, 86]. For example, a participant from an American/Australian sample with “EUPD” avoided healthy long-term relationships by purposely involving herself with men who she felt were the *“wrong type of guys”* because she was clear it was *“not going anywhere”* [86]. Similarly, some participants with an “EUPD” diagnosis/traits tested people for possible closeness and relationships to see *“whoever can cope”* and therefore if the individual could be trusted [76, 86].

Other participants masked their fears when socializing with others by finding ways of maintaining distance whilst simultaneously blending in [36, 73, 87]. For instance, participants with a diagnosis of “AVPD” described always feeling on guard against potential danger [69, 73]. They explained that they would attempt to prevent being noticed by wearing sunglasses to avoid eye contact, withdrawing from social situations by staying quiet and hiding “behind others whom they perceived to be somewhat safe” and “better able to master most social situations” [69, 73].

Conversely, a minority of studies emphasized that participants tried to act as if normal to hide and conceal “their perceived shortcomings” [67, 73, 76]. Participants from a Norwegian sample with a diagnosis of “AVPD” [73], a Swedish sample of women with a diagnosis/traits of “EUPD” [76], and a UK sample with “personality disorder” [67] described *“putting on a mask”.* This was mentioned in the context of their attempts to avoid the stigma associated with “personality disorder”, by following templates for appropriate behaviours in a given situation:

*“When I’m with H I’m collected and controlled. I always am when I’m together with other people. Inside I’m not one dammed bit controlled, just have such anxiety”* (Interviewee from a Swedish sample diagnosed with “EUPD”) [76]

Some participants, largely with “AVPD” and “EUPD” diagnoses preferred to withdraw from social situations by physically disconnecting, resulting in increased feelings of loneliness [36, 66, 73]:

*“when times are tough I go inside myself and I retreat from the world and I hide in my house and this is where the loneliness comes in I suppose.”* (Interviewee from a UK sample with a diagnosis of “EUPD”) [36].

### Theme 5: experiences of existential loneliness

Some narratives described the experience of being cut off and unable to engage in the social world resulting in a feeling of purposelessness. This appeared to be a feature of what was experienced as a meaningless and empty life. Specifically, this theme reflected a perceived lack of purpose in life or existential loneliness, which was described by participants in the context of a lack of collective purposeful activities and individual goals. This seemed to engender a sense of frustration, uselessness, emptiness, and social exclusion. People from UK samples [36, 70, 90], an Australian [74] and a French, Belgium, and Swiss sample of adolescents [72], primarily those with a diagnosis of “EUPD”, described a feeling of solitude and lack of engagement in leisure activities, which induced feelings of hopelessness, withdrawal, and a form of *“soul-destroying”* loneliness, in which individuals felt as though they had to drag themselves through every day [91].

*“I guess in a way it’s loneliness that I don’t have something planned or a bit of frustration that I can’t get myself going to do something*” (Interviewee from an Australian sample diagnosed with “EUPD”) [74]

This theme of a meaningless life, lacking in stimulating activities or social connections, appeared to be associated with difficulties establishing a stable and rooted way of life. Often this related to loss or lack of a job, geographical distance from others or itinerant living arrangements, and frequent or long periods of hospitalization [70, 72, 79, 90, 92]:

*“Yeah it gets worse* (the loneliness)*. It gets to the point where I’m thinking “oh I’m useless”, “I’m a nobody”, and none of my friends have phoned me to see how I am, so obviously no one cares.”* (Interviewee from an Australian sample diagnosed with “EUPD”) [74]

*“It was quite a shock when I went home after being on the ward for 4 months, living alone: as much as I like peace and quiet, it’s deathly quiet and weird after the ward: I underestimated how lonely and quiet it was going home”* (Interviewee from a UK sample diagnosed with “personality disorder” and mood disorders) [92]

### Theme 6: recovery, embedded in a social world

Participants with a diagnosis/traits of “personality disorder” in a range of studies described how important a sense of belonging and integration was for personal recovery, maintaining wellbeing, and crisis management [61, 62, 64, 67, 68, 77, 81, 82, 85, 89, 93–97]:

*“For me personally it means … sort of reintegration into the community and sort of mainstream society … to combat the feeling of alienation that I experience*” (Interviewee from a UK sample with a diagnosis/self-reported “personality disorder”) [67]

Participants hankered after a sense of group belonging in which they finally felt understood and heard, and were united by a common purpose. This was viewed as a *“dream come true”*, especially for participants with a diagnosis/traits of “EUPD” [61, 64, 67, 77, 82, 89, 94, 95]. Despite participants emphasizing the importance of relationships with friends and family, this sense of belonging was usually developed in a therapeutic group setting surrounded by “like-minded people”. This allowed participants to foster deep connections in a safe place through their ability to identify with each other and feel “normal”, thus reducing levels of loneliness and the sense of otherness [61, 62, 64, 67, 77, 81, 94–97]:

*“It was good knowing that there was other people out there … it got rid of that kind of loneliness and am I a freak?”* (Interviewee from a UK sample diagnosed with “EUPD”) [97]

Participants from UK and Australian samples with “EUPD” explained that group therapy was like finding a family they never had “under one roof”, suggesting that forming deep connections and feeling a sense of integration were important yet rare experiences pivotal to recovery, unattainable in mainstream society [77, 82, 94]:

*“I don’t feel like a weirdo … it’s actually almost nurturing for me … This is like almost an adopted family for me … I can actually feel myself doing all the learning that perhaps I should have been doing donkeys years ago”* (Interviewee from a UK

sample diagnosed with “EUPD”) [82]

In UK [61, 64, 82, 90, 96], Dutch [89], Canadian [95], and American [97] samples, participants reported that taking part in a group with shared understandings regarding the experience of “personality disorder” appeared to alleviate the symptoms of “personality disorder” by restoring hope in others, re-establishing a sense of inclusivity and identity amongst others, and removing the “loneliness of living with the painful emotions”. This was particularly emphasized by participants with “EUPD”. Participants described having gained the ability to “feel less paranoid of others”, had developed a balanced view of others, felt supported to confront deeply-ingrained fears, and had formed desired connections, all of which had reduced their sense of exclusion and loneliness [64, 79, 89, 90, 95, 97].

### Theme 7: group therapy: a setback

We identified a small group of studies that highlighted individuals’ preference to psychologically withdraw and distance themselves from others with similar difficulties, as encountered in group therapy [62, 64, 70, 71, 80, 90, 95]. For the individuals described, group therapy was perceived as unsupportive and as impeding their journey towards recovery. Indeed, for some, the idea of engaging in a therapeutic group was viewed as a potentially harmful setback in their journey, especially when feeling fragile [67, 73, 94].

*“It’s difficult when you’re dealing with other people who have a lot of the same problems … sometimes it feels like this is a new identity; that this is who you are; you are part of this group of people who have these problems and sometimes that’s a bit hard to do”* (Interviewee from a UK sample with a diagnosis of “EUPD”) [82]

Connected with subtheme 4.1 (A rejecting and hostile external world), participants from a UK sample with “personality disorder” diagnoses/traits perceived that being in a therapeutic community setting could trigger fears of rejection and judgment by others which would negatively impact their recovery progress [67]:

*“I think if you’re put in a situation where people would get to know me, I think that could have a negative effect on my recovery and not a positive* one” [67]

Being in a group and seeing group members joining and making friends was sometimes experienced as reinforcing a sense of otherness and alienation [93]:

*“Sometimes I do feel alone at the groups. I struggle with this idea of community*.” (Interviewee from a UK sample with a diagnosis of “personality disorder”) [93]

Participants from some UK samples described negative aspects of being in a group such as a sense that support from other group members in therapy, for “EUPD”, was “lacking or inconsistent” [65, 85]. Moreover, participants diagnosed with “EUPD” also found it disheartening to see themselves “as belonging to a group of people with problems” [82].

## Discussion

### Main findings

Our synthesis analysis of 39 qualitative studies describing experiences of loneliness among people with a “personality disorder” diagnosis/traits identified seven over-arching themes, all reflecting experiences of loneliness as a central part of the difficulties faced by this group of people.

Theme 1 suggested that people with a diagnosis/traits of “personality disorder”, particularly “EUPD” and “AVPD”, experienced intense and persistent alienation, disconnection and feelings of emptiness, not necessarily alleviated by having social contacts. Core features of chronic emptiness, such as an intense sense of disconnection from others, a sense of deadness, and feeling invisible to others, that seemingly contributed to self-harm, were described alongside feelings of social disconnection and loneliness. As indicated by Theme 2, early alienating and rejecting childhood experiences were often reported to cause feelings of disconnection and loneliness. A desire to connect and form ‘real’ relationships was evident in Theme 3, however people with a diagnosis/traits of “EUPD” and “AVPD” conveyed a simultaneous fear of closeness, as described in Theme 4. This appeared to arise in part from experiences of stigma and rejection in a range of contexts; these experiences engendered perceptions of a rejecting and threatening social world that individuals felt they must retreat from to maintain safety. People with a diagnosis/traits of “personality disorder” actively combated these intense paradoxical feelings by employing self-management strategies; for instance, people with a diagnosis of “AVPD” developed approaches to blend in by either hiding behind others or masking insecurities, whilst people with a diagnosis/traits of “EUPD” and “AVPD” described compensating for lack of human connections via connections with pets.

Theme 5 described experiences of a meaningless and empty life, capturing a perceived lack of shared leisure activities and goals, thus reinforcing experiences of existential loneliness. Theme 6 suggested that, for many, achieving a sense of belonging and feeling less lonely was an essential component of recovery. This was particularly apparent for people with a diagnosis/traits of “EUPD”, for whom a sense of belonging reduced painful feelings of alienation and otherness. Group therapy was frequently described as a route to recovering from early conflictual family relations, and from the resulting feelings of differentness and rejection. Effects of belonging to other kinds of groups were less explored. However, as described in Theme 7, a minority of studies described some individuals’ preference to withdraw or avoid the group therapy components of their treatment due to a fear of group dynamics reinforcing their sense of alienation. Throughout these data, it seemed that the relationship between loneliness and “personality disorder” was mutually reinforcing. In this feedback loop core symptoms and processes of “personality disorder”, such as disturbed attachments, led to unmet social needs. In turn, these paved the way for experiences of loneliness, which further exacerbated “personality disorder” symptoms.

### Findings in the context of other studies

Our theme regarding origins of loneliness in childhood alienation is congruent with the findings of several quantitative studies and reviews on “personality disorder” that link disturbed attachment and perceived parental rejection with subsequent loneliness experiences and the perception of others as rejecting and malevolent [39, 46, 98–100]. This is consistent with the literature describing an association between loneliness and trauma [101, 102]; and between “personality disorder” (particularly “EUPD”) and early traumatic life experiences [103, 104]. Additionally, professionals can also play a role in reinforcing past rejecting and traumatic experiences that intensify loneliness experiences. For example, a recent qualitative thematic synthesis of qualitative literature on service user views reported that pessimistic and stigmatising views were frequently accounted among clinicians in mental health services [21]. Many clinicians, especially in generic mental health services, where perceived as lacking the compassion and the knowledge needed to support people with a diagnosis of “personality disorder”, contributing to service users feeling isolated and marginalized” [21].

The simultaneous yearning for, yet fear of, connection can be understood in the context of disrupted attachments in which caregivers are both a source of threat yet also security. This was described by Holmes as ‘approach-avoidance dilemmas’ which characterize the relationship and attachment between infants and caregivers for people with “EUPD” diagnosis [105].

Studies exploring group identification interventions, that focus on improving social group ties and building group-based social identification, further support our findings that the intensity of social disconnection experienced can potentially be alleviated through the sense of belonging and identity established in group therapy settings, as opposed to mere social contact [106, 107]. In line with the Social Identity Theory, evaluations of two interventions, Groups 4 Health and the Community Navigator intervention, have indicated that promoting socially focused group engagement may address social disconnection and loneliness [106, 108]. Specifically, the evaluation of the Group 4 Health intervention for adults experiencing social isolation, depression, and anxiety found improvements in loneliness that were underpinned by changes in social identification and group belonging [106]. People diagnosed with “personality disorder” experiencing loneliness may therefore benefit from similar interventions, particularly as the Community Navigator intervention targets some issues and symptoms that overlap with “personality disorder”.

A meta-synthesis exploring the experiences of people with “EUPD” on acute psychiatric inpatient wards found that contact with other patients with similar difficulties was perceived to be beneficial [109]. However, one study of UK service users diagnosed with “EUPD” found that relationships perceived as unhelpful can hinder recovery and wellbeing, occasionally contributing to discontinuation of therapy, and therefore, being in a group setting that is perceived as threatening is likely to be detrimental [82].

Similar to our findings, cross-sectional quantitative research indicates that insecure attachment is associated with perfectionistic self-presentation, such as masking insecurities and hiding imperfection, as a means towards social acceptance and forming connections [110]. Similarly, facets of perfectionistic self-presentation are found to be associated with social disconnection and alienation [110]. These findings align with the perfectionism social disconnection theory, which postulates that early rejecting experiences give rise to unfulfilled social needs, and this motivates individuals to mask insecurities and present a “flawless façade” to avoid rejection [110]. Hyper-vigilance to, and expectancies of, rejection and perfectionistic self-presentation may further create rejecting, hostile and alienating experiences [110–112], thus, potentially contributing to a “self-fulfilling prophecy feedback loop” that perpetuates loneliness experiences [11]. This self-fulfilling prophecy was also apparent in a recent meta-synthesis exploring the experience of loneliness in young people with depression, which also identified themes describing a desire for connections, yet an inability to tolerate others and a fear of rejection [11].

Although loneliness and chronic emptiness are distinctive constructs [51], our findings regarding the construct of chronic emptiness indicated that difficulties associated with emptiness overlap with difficulties that result from loneliness experiences. As captured in theme 1, we found that loneliness and the intense sense of disconnection reported was also described alongside core features of chronic emptiness such as a sense of deadness, nothingness, and invisibility. This suggested that there are some commonly shared difficulties associated with both loneliness and emptiness. According to our themes and previous research, a sense of deadness, a commonly reported emotion among people who report feeling lonely and empty, is associated with feelings of disconnection which may sometimes feature when engaging in self-harm [50, 51]. It also appears that theme 5 (Experiences of Existential Loneliness) shares similarities with experiences that are commonly associated with emptiness. Narratives in theme 5 describing a lack of purpose and meaningful life overlap with cognitions and emotions of purposelessness and unfulfillment that are associated with chronic emptiness [49]. Previous qualitative research studies and systematic reviews on chronic emptiness and loneliness has emphasized that emptiness is a distinct experience, which is largely defined as a disconnection from the self, and to a lesser degree, a sense of disconnection from others, with a sense of an unfillable inner void [49–51, 113]. The distinguishing factor between these constructs is that whilst emptiness is conceptualized as an absence of feelings and a personal form of disconnection from the self, loneliness is described as a feeling of social disconnection from others and the world that potentially precede emptiness experiences [50]. To further demonstrate the distinction, theoretical explanations of emptiness have suggested that emptiness serves a defensive function in response to potentially profound suffering resulting from trauma or an inability to hold a stable image of the self; a difficulty that is closely associated to a sense of deadness [49, 114]. In light of this conceptualization of emptiness, it is possible that our data from theme 1 capture the initial feelings of social disconnection and loneliness as well as later sequelae, namely the exacerbation of feelings of emptiness that can result from the painful experience of loneliness.

#### Strengths and limitations

We used a well-established approach to synthesizing qualitative findings from samples of people with “personality disorder” [57]. Our search terms were broad, including concepts relating to loneliness, thus providing us with a rich dataset describing loneliness-related experiences. Issues of reflexivity were addressed through regular meetings of the multidisciplinary review team of academics and clinicians, with experience of conducting reviews exploring loneliness experiences. Our inclusion criteria were pragmatic, in that we included research involving patients with “personality disorder” and other psychiatric comorbidities, reflecting clinical reality [18, 115].

Despite some cultural diversity, we acknowledge that a major limitation of this meta-synthesis is the limited range of perspectives included particularly the lack of lived experience researcher perspective on our team. The findings of this meta-synthesis are also limited by the methodological quality of included studies, where a key weakness was a failure of some authors to consider reflexivity. We also acknowledge issues of generalizability, as the majority of studies included in this meta-synthesis explored loneliness experiences in women with “EUPD”. This is a focus with clinical relevance, as this group is highly represented in specialist “personality disorder” services. Additionally, there is inconsistency between studies in whether they also include any other groups of “personality disorder”, and the conclusions we have drawn are likely to be principally applicable to people with Cluster B diagnoses. This is especially a limitation as our themes do not capture or distinguish between possible differences in the experiences of loneliness faced by individuals along the spectrum of difficulties associated with “personality disorder” traits/diagnoses. We also acknowledge that, as discussed above, the concept of “personality disorder” is a contested one, a limitation relevant across the literature we have reviewed. Other restrictions on representativeness are that people agreeing to take part in the included studies may differ from those not recruited, and the studies were mainly conducted in higher income countries, especially the UK. Some studies did not report on participant demographics or specify the type of “personality disorder”, which limited our ability to explore patterns of themes by gender, ethnicity, and age. Moreover, given the range of settings and methodological approaches employed in the included studies, the context of each study was relatively neglected, which may contribute to more superficial interpretations. Only two included studies had exploring loneliness experiences in people with “personality disorder” as their primary goal [36, 42] thus, we relied on relevant material extracted from broader work exploring experiences of social relationships. Furthermore, despite the high prevalence of loneliness among people within the criminal justice system, our themes on loneliness cannot be assumed to generalize to this population.

#### Clinical implications and future research

This meta-synthesis demonstrated the centrality and intensity of loneliness in the lives of people with a diagnosis/traits of “EUPD”, as well as a complex vicious cycle, in which loneliness and difficulties in managing interpersonal relationships are mutually reinforcing. This suggests a need to develop, in collaboration with people with relevant lived experience, tailored interventions, particularly socially-focused interventions that target experiences of loneliness. Particularly, socially focused interventions that target one’s sense of self through social group membership and principles of Social Identity Theory could potentially alleviate feelings of loneliness and indirectly target emptiness [108]. There are indications, not yet clearly demonstrated in trial evidence, that changing cognitions may reduce loneliness in people with mental health problems [43]. Our findings regarding the persistent and enduring alienation from others experienced by people with a “personality disorder” diagnosis/traits suggest that such psychological approaches could be beneficial. Other potential approaches to reducing loneliness in people with mental health problems are more directly focused on supporting people in trying to form and sustain meaningful connections [43]. For instance, strategies developed for groups with overlapping difficulties associated with “personality disorder” include the Community Navigator approach tailored to people with severe depression and anxiety and Groups 4 Health intervention targeted for people with psychological distress arising from social isolation offer a potential promising therapeutic focus [108]. Peer support is another potential approach. Our findings suggest considerable potential benefits from developing a sense of belonging within a therapeutic group. This suggests a need to evaluate peer support schemes in delivering such benefits.

Further research is needed to ascertain which approaches are acceptable and feasible for people with “personality disorder” diagnoses/traits, as a prelude to further development, adaptation and testing. Pending this, evidence from our study and related quantitative work on the apparent centrality of loneliness for many people with a “personality disorder” diagnosis suggests that there are potential benefits to routinely asking service users about feelings of loneliness. This is in keeping with service user and clinician calls for a more holistic approach to supporting people with a “personality disorder” diagnosis in their day to day lives to improve quality of life and clinical outcomes [21].

Intervention development should be informed by further studies, including co-produced qualitative studies exploring specifically the experience of loneliness among people with “personality disorder”, as only two identified articles addressed this. Such studies should sample in order to understand how loneliness experiences vary in relation to ethnicity, sexuality, and other protected characteristics, as well as by specific “personality disorder” or spectrum of challenges associated with the traits and diagnosis of “personality disorder”. It would also be important for such work to probe the role of stigma in the context of loneliness and “personality disorder”, as well as explore experiences of friendships, and factors that contribute to perceptions of an unhelpful and harmful therapeutic group dynamic. All such qualitative research should include consideration of reflexivity to improve the rigour of findings regarding loneliness experiences. Moreover, future studies should consider exploring the role of loneliness among people with “personality disorder” traits/diagnoses within the criminal justice system as social disconnection and loneliness experiences may be complex and shaped by distinctive prison-related experiences [116].

## Conclusion

This meta-synthesis of 39 qualitative studies derived 7 themes describing experiences of loneliness among people with a “personality disorder” diagnosis or traits. Among these themes, it was apparent that enduring feelings of loneliness among people with “personality disorder” diagnoses/traits often arises from early rejecting familial experiences. Participants described how these early experiences created unmet social needs for social connectedness, which seemed to contribute to future experiences of disconnection and loneliness. A self-fulfilling feedback loop in which participants anticipated threatening and rejecting experiences seemed to perpetuate loneliness. For some people, group therapy helped promote a sense of belonging and understanding that alleviated loneliness. The centrality of loneliness in the lives of people with “personality disorder” diagnoses/traits suggest a need to develop and trial interventions to break the cycle of loneliness and social difficulties.

## Supplementary Information

**Additional file 1.**

**Additional file 2.**

**Additional file 3.**

## Data Availability

All data is published and is under the public domain.

## Abbreviations

EUPD: Emotionally unstable personality disorder
AVPD: Avoidant personality disorder
CASP: Critical Appraisal Skills Program
BPD: Borderline personality disorder
SD: Standard deviation
n: Number of participants
F: Females
UK: United Kingdom
