# Supplementary material for: The experience of loneliness among people with a “personality disorder” diagnosis or traits: a qualitative meta-synthesis
Source: BMC Psychiatry. 2022 Feb 17;22:130. doi: 10.1186/s12888-022-03767-9 (PMC8855579; doi:10.1186/s12888-022-03767-9)
Supplement: Supplementary file 1 — Additional file 1. [file 12888_2022_3767_MOESM1_ESM.docx]

Supplementary Table. 1. *Table presenting characteristics and quality appraisal of eligible articles*

| Citation | Study setting | Study aims | Sample size N, diagnosis or self-reported or traits,  type of personality disorder | Sample characteristics: Mean age (SD) or Median, age range, gender (female n), ethnicity | Data collection method | Data analysis method | Major Themes | CASP Score |
| --- | --- | --- | --- | --- | --- | --- | --- | --- |
| **Agnew et al., 2016**  **Self and identity in women with symptoms of borderline personality: A qualitative study** | Multidisciplinary community mental health teams, UK | To explore the concept of self/identity from the individual’s perspective | N= 5  A working diagnosis of borderline personality disorder | Aged 30-45 years  F= 5 | Life story interviews | Thematic analysis | 1) Connecting to myself  2) Distance between us  3) Hurt and healing | Clear aims (2)  Appropriate qualitative method (2)  Appropriate research design (2)  Appropriate recruitment strategy (2)  Appropriate data collection (2)  Consideration for reflexivity (0)  Consideration of ethical issues (2)  Sufficiently rigorous data analysis (2)  Clear statement of findings (2)  Value of research (2) |
| **Borschmann et al.,**  **(2014)**  **Advance Statements for Borderline Personality Disorder: A Qualitative Study of Future Crisis Treatment Preferences** | Community mental health teams within South London, UK | To investigate crisis treatment preferences of a sample of community-dwelling adults with borderline personality disorder | N=41  Diagnosed with borderline personality disorder | Mean age (SD): 35.6 years (11.0)  F=34  White= 32  Black= 5  Asian= 2  Others= 2 | Open structured discussion meetings with participant. Participant permitted to bring a community mental health team clinician, caregiver, friend, other health professional, or other advocate | Thematic analysis | 1) Experience of crisis  2) Connecting with or disconnecting from others in a crisis  3) Exacerbating factors  4) Interactions with mental health  professionals during crises  5) Specific refusals regarding treatment  6) Dissemination of joint crisis plans | Clear aims (2)  Appropriate qualitative method (2)  Appropriate research design (2)  Appropriate recruitment strategy (2)  Appropriate data collection (1)  Consideration for reflexivity (0)  Consideration of ethical issues (1)  Sufficiently rigorous data analysis (2)  Clear statement of findings (2)  Value of research (2) |
| **Birkens & Harper., (2017)**  **Experiences of people with a personality disorder or mood disorder regarding carrying out daily activities following discharge from hospital** | South West London,  outpatient departments of the Community Mental Health Teams of the NHS trust, UK | To explore the experiences of people with a personality or mood disorder regarding carrying out everyday activities following discharge from hospital | N= 16  7 with a diagnosis of personality disorder; 7 with a diagnosis of mood disorder; 2 participants had both diagnoses. | Age: Not stated  F= 11  White British= 14  Black British= 1  British Asian= 1 | Semi-structured interviews | Thematic analysis | 1) The cumulative eﬀect of multiple admissions on activities of daily living.  2) The consequences of long admissions on daily life.  3) Social isolation reducing activities in the ﬁrst few months’ post-discharge.  4) Activities that people would like to do better, post-discharge from hospital  5) Longer term goals | Clear aims (2)  Appropriate qualitative method (2)  Appropriate research design (2)  Appropriate recruitment strategy (2)  Appropriate data collection (1)  Consideration for reflexivity (2)  Consideration of ethical issues (2)  Sufficiently rigorous data analysis (2)  Clear statement of findings (2)  Value of research (2) |
| **Bradley-Scott, 2017**  **Exploring mentalization-based psychoeducation groups for people with borderline personality disorder** | Secondary-care services in the South-East of  England, UK | To explore lived experiences of manualised mentalization-based psychoeducation groups for people with borderline personality disorder | N=8  Diagnosed with borderline personality disorder | Aged 20-45 years  F= 5  White= 8 | Semi-structured interviews | Interpretative phenomenological analysis | 1) Managing complex group processes  2) Increased understanding: power and fear  3) Personalising knowledge | Clear aims (2)  Appropriate qualitative method (2)  Appropriate research design (2)  Appropriate recruitment strategy (2)  Appropriate data collection (2)  Consideration for reflexivity (1)  Consideration of ethical issues (2)  Sufficiently rigorous data analysis (2)  Clear statement of findings (2)  Value of research (2) |
| **Black et al., 2013**  **What do people in forensic secure and community settings think of their personality disorder diagnosis? A qualitative study** | Forensic medium  secure and community services in South London, UK | To explore the experience of having a personality disorder diagnosis within the context of forensic secure and community services | N=10  Diagnosed with a personality disorder | Age: Not stated  F=2 | Interviews | Interpretative phenomenological analysis | 1) How I am now (the person and their life)  2) A difﬁcult diagnosis (the personality disorder label and its consequences). | Clear aims (2)  Appropriate qualitative method (2)  Appropriate research design (2)  Appropriate recruitment strategy (2)  Appropriate data collection (1)  Consideration for reflexivity (0)  Consideration of ethical issues (2)  Sufficiently rigorous data analysis (2)  Clear statement of findings (2)  Value of research (2) |
| **Castillo et al., 2013**  **A recovery journey for people with personality disorder** | The Haven, therapeutic community and crisis house, Colchester, Essex, UK | To explore what recovery means to people with personality disorder; develop a conceptual model of recovery in personality disorder; evaluate the contribution of the setting to recovery practice | N=60  Diagnosed with personality disorder | Aged 18-65 years  F= 47  White British= 57  British-born Pakistani= 1  Dutch= 1  White American – USA= 1 | Focus groups, individual interviews and background data collected at the service | Thematic analysis | 1) A sense of safety and building trust  2) Feeling cared for and creating a culture of warmth  3) A sense of belonging and community  4) Learning the boundaries – love is not enough  5) Containing experiences and developing skills  6) Hopes, dreams and goals and their relationship to recovery  7) Achievements, identity and roles  8) Transitional recovery and how to maintain healthy attachment | Clear aims (2)  Appropriate qualitative method (2)  Appropriate research design (2)  Appropriate recruitment strategy (2)  Appropriate data collection (2)  Consideration for reflexivity (0)  Consideration of ethical issues (1)  Sufficiently rigorous data analysis (2)  Clear statement of findings (2)  Value of research (2) |
| **Clarke, 2017**  **The case for "fluid" hierarchies in therapeutic communities** | Democratic therapeutic community (TC), UK | To explain how therapeutic community client members negotiated and enforced community expectations through an analysis of power within everyday interactions outside of structured therapy | N=Not stated  Diagnosis of personality disorder | Age/gender: Not stated | Narrative ethnography consisting of participant observation and narrative interviews | Thematic analysis | 1) Empowerment through inclusion  2) Power through exclusion | Clear aims (2)  Appropriate qualitative method (2)  Appropriate research design (1)  Appropriate recruitment strategy (1)  Appropriate data collection (1)  Consideration for reflexivity (0)  Consideration of ethical issues (2)  Sufficiently rigorous data analysis (1)  Clear statement of findings (1)  Value of research (2) |
| **Clarke & Waring, 2018**  **The transformative role of interaction rituals within therapeutic communities** | Therapeutic community, UK | To explain how everyday social interactions outside of formal therapy can facilitate transformative  personal change. | N=Not stated  Diagnosis of personality disorder | Age/gender: Not stated | Narrative ethnography Participant observation and narrative interviews | Interpretative data analysis | 1) Inclusivity within rituals: solidarity through negative transient emotions  2) Transforming negative transient emotions in to high EE  3) Exclusivity within rituals: negative transient emotions reinforcing low EE | Clear aims (2)  Appropriate qualitative method (2)  Appropriate research design (2)  Appropriate recruitment strategy (1)  Appropriate data collection (1)  Consideration for reflexivity (0)  Consideration of ethical issues (2)  Sufficiently rigorous data analysis (1)  Clear statement of findings (1)  Value of research (2) |
| **Cunningham et al., (2004).**  **It's About Me Solving My Problems: Clients' Assessments of Dialectical Behavior Therapy** | Assertive community treatment team, Kalamazoo, Michigan, US | To understand, from the perspective of the client, what is effective about DBT and why | N= 14  Diagnosed with borderline personality disorder | Mean age: 38.7 years  aged 23-61 years  F= 14 | Open-ended, semi-structured interviews | Interpretative analysis | 1) General reflection  2) Assessment of Program components  3) Effect of DBT on day-to-day life | Clear aims (2)  Appropriate qualitative method (2)  Appropriate research design (2)  Appropriate recruitment strategy (2)  Appropriate data collection (1)  Consideration for reflexivity (1)  Consideration of ethical issues (1)  Sufficiently rigorous data analysis (2)  Clear statement of findings (2)  Value of research (2) |
| **Donald et al., 2017**  **Consumer perspectives on personal recovery and borderline personality disorder** | Specialist borderline personality disorder outpatient service in Australia | To explore how recovery occurs from the perspective of consumers | N=17  Diagnosed with borderline personality disorder | Mean age: 33.9 years  Aged 19-59 years  F=15 | Semi-structured interviews | Grounded theory and thematic analysis | 1) Understanding recovery  2)Conditions of change | Clear aims (2)  Appropriate qualitative method (2)  Appropriate research design (2)  Appropriate recruitment strategy (2)  Appropriate data collection (1)  Consideration for reflexivity (0)  Consideration of ethical issues (1)  Sufficiently rigorous data analysis (2)  Clear statement of findings (2)  Value of research (2) |
| **Dyson & Brown 2016**  **The experience of mentalization-based treatment: an interpretative phenomenological study** | Mentalization-based treatment programme, UK | To explore the experience of mentalization-based treatment in order to understand the significance, meaning, and quality this therapeutic intervention holds for them | N=6  Diagnosed with  borderline personality disorder | Aged 20-44 years  F= 6 | Semi-structured interviews | Interpretative phenomenological Analysis | The Battle between BPD and Me:  1) I’m much better now [Laughs]. Hopefully  2) You’ve got to be ready for therapy...You’ve got to be able to change.  3) We are one (but not together) | Clear aims (2)  Appropriate qualitative method (2)  Appropriate research design (2)  Appropriate recruitment strategy (2)  Appropriate data collection (1)  Consideration for reflexivity (2)  Consideration of ethical issues (2)  Sufficiently rigorous data analysis (2)  Clear statement of findings (2)  Value of research (2) |
| **Falklöf & Haglund., 2010**  **Daily Occupations and Adaptation to Daily Life Described by Women Suffering from Borderline Personality Disorder** | Outpatient  program in Southern part of Sweden | To examine how women with borderline personality disorder describe their daily occupations and adaptation to daily life | N=9  Diagnosed with borderline personality disorder | Mean age: 26.0 years  Aged 20-39 years  F= 9 | Semi-structured interviews: including the Occupational Circumstance Assessment—Interview and Rating Scale | Content analysis | 1) Performance  2) Self-image | Clear aims (2)  Appropriate qualitative method (2)  Appropriate research design (2)  Appropriate recruitment strategy (2)  Appropriate data collection (2)  Consideration for reflexivity (2)  Consideration of ethical issues (1)  Sufficiently rigorous data analysis (2)  Clear statement of findings (2)  Value of research (2) |
| **Frommer et al., 1996**  **Subjective Theories of Illness in Patients with Personality Disorders: Qualitative Comparison of Twelve Diagnostic Interviews** | England, UK | To describe core features of the patients’ narratives in terms of their subjective theories of illness | N=9  Diagnosed with a personality disorder  (Five had were diagnosed with borderline personality disorder, three with narcissistic personality disorder, one with histrionic personality disorder, one with schizoid personality disorder, one with paranoid personality disorder, and one with schizotypical personality disorder) | Mean age: 30.8 years  Aged 19-39 years  F=7 | Interviews | Content analysis | 1) Subjective theory of illness  2) Patients history  3) Personality traits | Clear aims (2)  Appropriate qualitative method (2)  Appropriate research design (2)  Appropriate recruitment strategy (1)  Appropriate data collection (1)  Consideration for reflexivity (1)  Consideration of ethical issues (1)  Sufficiently rigorous data analysis (2)  Clear statement of findings (2)  Value of research (0) |
| **Gilbert et al., 2013**  **“I Don’t Want to Live Like This Anymore”: Disrupted Habitus in Young People “At Risk” of Diagnosis of Personality Disorder** | Early intervention service , UK | To provide a sociological analysis to highlight links between individual experiences and the broader social context; suggest ways these insights may inform interactions with similarly positioned young people in the future | N=27  At risk of personality disorder based on assessment and signs of personality disorder  (type of assessment was not stated) | Aged 16-25 years  F=17  White British= 26 | Interviews | Narrative analysis | 1) How Young People Interpret Their Lives: “I Don’t Want to Live Like This Anymore”  2) What Young People Say About Their Lives: Dangerous and Troubled Relationships, Isolation, and Little Support | Clear aims (2)  Appropriate qualitative method (2)  Appropriate research design (2)  Appropriate recruitment strategy (2)  Appropriate data collection (1)  Consideration for reflexivity (0)  Consideration of ethical issues (2)  Sufficiently rigorous data analysis (1)  Clear statement of findings (1)  Value of research (2) |
| **Gillard et al., 2015**  **Understanding recovery in the context of lived experience of personality disorders: a collaborative, qualitative research study** | Specialist personality disorders service in London, UK | To explore understandings of recovery from the perspectives of people with lived experience of personality disorders | N= 6  Self-reported or diagnosis of personality disorder: (3 participants with borderline personality disorder, 1 participant with avoidant personality disorder, 2 participants did not specify the type of diagnosis) | Aged 26-65 years  F=3  White= 5 | In-depth interviews | Thematic and framework analysis | 1) The lived experience of personality disorders  2) Personality disorders and recovery  3) Treatment and support | Clear aims (2)  Appropriate qualitative method (2)  Appropriate research design (2)  Appropriate recruitment strategy (2)  Appropriate data collection (2)  Consideration for reflexivity (2)  Consideration of ethical issues (2)  Sufficiently rigorous data analysis (2)  Clear statement of findings (2)  Value of research (2) |
| **Holm & Severinsson., 2010**  **Desire to survive emotional pain related to self-harm: A Norwegian hermeneutic study** | Variety of settings in West coast of Norway | To explore and interpret women’s desire to survive emotional pain related to self-harm | N=13  Borderline personality disorder (whether diagnosed or self-reported is not specified) | Mean age: 39.0 years  Aged 25-53 years  F=13 | In-depth interviews | Interpretative hermeneutic approach | 1) Self-sacrifice  - Self-Harm: a struggle to be relieved of responsibility  - Fear of intimacy versus intrusion | Clear aims (2)  Appropriate qualitative method (2)  Appropriate research design (2)  Appropriate recruitment strategy (1)  Appropriate data collection (1)  Consideration for reflexivity (2)  Consideration of ethical issues (2)  Sufficiently rigorous data analysis (2)  Clear statement of findings (2)  Value of research (2) |
| **Johnson et al., 2016**  **Mentalizing after mentalization based treatment** | Secondary care in NHS, London, UK | To explores what three people experienced immediately after they completed an intensive mentalization-based treatment program | N= 3  Personality disorder (whether diagnosed or self-reported was not specified) | Age/gender: Not stated | Timelines and repeated cycles of audio-recorded focus groups | Thematic analysis | 1) Pre-MBT  2) End of MBT  3) At point of time-line | Clear aims (2)  Appropriate qualitative method (2)  Appropriate research design (2)  Appropriate recruitment strategy (2)  Appropriate data collection (1)  Consideration for reflexivity (0)  Consideration of ethical issues (1)  Sufficiently rigorous data analysis (2)  Clear statement of findings (1)  Value of research (2) |
| **Juurlink et al., 2019**  **Barriers and facilitators to employment in borderline personality disorder: A qualitative study among patients, mental health practitioners and insurance physicians** | Outpatient clinic, Netherlands | To explore the barriers and facilitators of gaining and maintaining employment in borderline personality disorder in patients, mental health practitioners (MHPs) and insurance physicians (IPs) | N= 15  Diagnosed with borderline personality disorder | Mean age: 39.0 years  Aged 23-58 years  F=14 | Semi-structured interviews and focus groups | Thematic content analysis | 1) Characteristics of BPD  2) Stigma  3) Support to employment | Clear aims (2)  Appropriate qualitative method (2)  Appropriate research design (1)  Appropriate recruitment strategy (2)  Appropriate data collection (2)  Consideration for reflexivity (1)  Consideration of ethical issues (1)  Sufficiently rigorous data analysis (2)  Clear statement of findings (2)  Value of research (1) |
| **Katsakou et al., 2012**  **Recovery in Borderline Personality Disorder (BPD): A Qualitative Study of Service Users’ Perspectives** | Mental health services in East London, UK | To explore what people with a diagnosis of  borderline personality disorder view as recovery | N= 48  Diagnosed with borderline personality disorder | Mean age (SD): 36.5 years (10.38)  F= 39  White= 33  Black= 5  Asian= 10 | In-depth semi-structured interviews | Grounded theory and thematic analysis | 1) Personal goals and/or achievements during recovery  2) Balancing personal goals of recovery versus service targets  3) How recovered do people feel?  4) Problems with the word ‘recovery’ | Clear aims (2)  Appropriate qualitative method (2)  Appropriate research design (2)  Appropriate recruitment strategy (2)  Appropriate data collection (2)  Consideration for reflexivity (1)  Consideration of ethical issues (1)  Sufficiently rigorous data analysis (2)  Clear statement of findings (2)  Value of research (2) |
| **Katsakou, 2016**  **Processes of recovery from Borderline Personality Disorder (BPD): A qualitative study** | Specialist and generic secondary mental health services in London, UK | To explore how recovery in borderline personality  disorder is reached through routine or specialist treatment, as perceived mainly by service users, but also by therapists and relatives. | N=69  n (service users with personality disorder): 48  Diagnosed with borderline personality disorder | Personality disorder group mean age: 36.5 years  Aged 18-58 years  F= 39  White= 33  Black= 5  Asian= 10 | Semi-structured interviews | Thematic analysis | 1) Process of recovery  2) Challenges in therapy | Clear aims (2)  Appropriate qualitative method (2)  Appropriate research design (2)  Appropriate recruitment strategy (2)  Appropriate data collection (2)  Consideration for reflexivity (2)  Consideration of ethical issues (2)  Sufficiently rigorous data analysis (2)  Clear statement of findings (2)  Value of research (2) |
| **Lamph., 2018**  **Enhancing Understanding of the Experience of People with Common Mental Health Disorders and Co-Morbid Personality Disorder Traits Who Present to Primary Care IAPT Services** | 5 Boroughs Partnership NHS Foundation Trusts, Wigan and Leigh IAPT service, London, UK | To explore and understand patients’ needs and treatment experiences within IAPT services | N=22  Screened and scored at least 3 out of 8 using the Standardised Assessment of Personality – Abbreviated Scale (SAPAS) a screening tool for likely personality disorder | Aged 18-65 years  F=13  White British= 21 | Interviews | Framework analysis | 1) Process and Business  2) Needs  3) Treatment experience  4) What matters | Clear aims (2)  Appropriate qualitative method (2)  Appropriate research design (2)  Appropriate recruitment strategy (2)  Appropriate data collection (2)  Consideration for reflexivity (2)  Consideration of ethical issues (2)  Sufficiently rigorous data analysis (2)  Clear statement of findings (2)  Value of research (2) |
| **Lariviere et al., 2015**  **Recovery, as Experienced by Women with Borderline Personality Disorder** | Specialized personality disorder program, Quebec, Canada | To capture the experience of recovery in women with borderline personality disorder | N=12  Diagnosis of borderline personality disorder | Mean age (SD): 37.2 years (13.3)  Aged 23-63 years  F= 12 | Semi-structured interviews, realization of a collage and personal object | Thematic analysis | 1) Living with BPD  2) Dimensions of Recovery  3) Dimensions Related to the Person  4) Dimensions Related to the Environment  5) Dimensions Related to Occupation  6) Facilitators of Recovery  7) Facilitators Related to the Environment  8) Facilitators Related to Occupation  9) Obstacles to Recovery | Clear aims (2)  Appropriate qualitative method (2)  Appropriate research design (2)  Appropriate recruitment strategy (2)  Appropriate data collection (1)  Consideration for reflexivity (0)  Consideration of ethical issues (1)  Sufficiently rigorous data analysis (1)  Clear statement of findings (2)  Value of research (0) |
| **Lonargáin et al., 2017**  **Service user experiences of mentalisation-based treatment for borderline personality disorder** | Mentalisation-based treatment group within NHS trust, UK | To explore how adults with difficulties associated with borderline personality disorder experience intensive out-patient mentalisation-based treatment | N= 7  Accessing mentalisation-based treatment for borderline personality disorder (whether diagnosed or self-reported was not specified) | Aged 26-52 years  F= 5 | Semi-structured interviews | Interpretative phenomenological analysis | 1) Experiencing group MBT as unpredictable and challenging  2) Building trust: a gradual but necessary process during MBT  3) Putting the pieces together: making sense of the overall MBT structure  4) Seeing the world differently due to MBT: a positive shift in experience | Clear aims (2)  Appropriate qualitative method (2)  Appropriate research design (2)  Appropriate recruitment strategy (2)  Appropriate data collection (1)  Consideration for reflexivity (1)  Consideration of ethical issues (2)  Sufficiently rigorous data analysis (2)  Clear statement of findings (2)  Value of research (2) |
| **McSherry et al., 2012**  **Service user experience of adapted dialectical behaviour therapy in a community adult mental health setting** | Community adult mental health center, UK | To examine service users’ perspective on the effectiveness of an adapted dialectical behaviour therapy programme | N=8  Diagnosed with borderline personality disorder | Aged 32-55 years  F= 6 | Semi-structured interviews and focus group | Thematic analysis | 1) Evaluation of therapy  2) Treatment impact | Clear aims (2)  Appropriate qualitative method (2)  Appropriate research design (1)  Appropriate recruitment strategy (2)  Appropriate data collection (1)  Consideration for reflexivity (0)  Consideration of ethical issues (1)  Sufficiently rigorous data analysis (1)  Clear statement of findings (2)  Value of research (1) |
| **Miller, 1994**  **Borderline personality disorder from the patient’s perspective** | University of South Florida psychiatry center, US | To learn how patients with borderline personality disorder experience the disorder and its treatment | N=10  Diagnosed with borderline personality disorder | Aged 21-50 years  F=8  Anglo-saxon= 10 | Life history narrative interviews | Grounded theory | 1) Self-definition  2) Being in the world: estrangement, inadequacy, and despair  3) Coping strategies  4) The patient experience  5) Self disclosure and the research process | Clear aims (2)  Appropriate qualitative method (2)  Appropriate research design (2)  Appropriate recruitment strategy (2)  Appropriate data collection (2)  Consideration for reflexivity (0)  Consideration of ethical issues (0)  Sufficiently rigorous data analysis (1)  Clear statement of findings (1)  Value of research (2) |
| **Musa et al., 2019**  **Thesis**  **An exploration of psychotic-like experiences in Borderline Personality Disorder** | Mental health services in East of England, UK | To explore the nature of psychotic-like experiences in help-seeking young people with borderline personality disorder traits and how they are appraised and responded to | N=7  Borderline personality disorder traits as assessed by HYPE, scoring above 11, and receiving support from the borderline personality disorder trait treatment pathway | Aged 16-25 years  F=3  Transgendered male= 1  White British= 7 | Semi-structured interviews | Interpretative phenomenological analysis | 1) Description of experiences  2) Making sense of the experiences  3) Deterioration of sense of self and well-being  4) Managing and finding respite | Clear aims (2)  Appropriate qualitative method (2)  Appropriate research design (2)  Appropriate recruitment strategy (2)  Appropriate data collection (1)  Consideration for reflexivity (2)  Consideration of ethical issues (2)  Sufficiently rigorous data analysis (2)  Clear statement of findings (2)  Value of research (2) |
| **Narayanan & Rao, 2018**  **Personality Disorders in the Indian Culture: Reconsidering Self Perceptions, Traditional Society and Values** | Inpatient and outpatient departments of National Institute of Mental Health and Neuro Sciences Karnataka, India | To study individuals with personality disorders as they present to the clinical setting | N=40  n (personality disorder group) = 20    Diagnosis of personality disorder | Personality disorder group mean age (SD): 28.90 years (6.09)  F (personality disorder group) = 10 | Questionnaires, focus groups and in-depth interviews | Content analysis | 1)Mood states that impacted the self  2) Signiﬁcant life experiences  3) Role of family of origin and procreation  4) Value organization | Clear aims (2)  Appropriate qualitative method (2)  Appropriate research design (2)  Appropriate recruitment strategy (2)  Appropriate data collection (1)  Consideration for reflexivity (0)  Consideration of ethical issues (2)  Sufficiently rigorous data analysis (2)  Clear statement of findings (1)  Value of research (0) |
| **Perseius et al., 2005**  **To Tame Volcano: Patients with**  **Borderline Personality Disorder and Their Perceptions of Suffering** | Outpatient clinic, Southern Sweden | To investigate life situations, suffering, and perceptions of encounter with psychiatric care | N=10  Diagnosed with borderline personality disorder | Mean age: 27.0 years  Aged 22- 49 years  F= 10 | Narrative interviews and biographical material (dairies and poems) | Hermeneutic analysis | 1) Life on the edge  2) The struggle for health and dignity—a balance act on a slack wire over a volcano  3) The good and the bad act of psychiatric care in the drama of suffering | Clear aims (2)  Appropriate qualitative method (2)  Appropriate research design (2)  Appropriate recruitment strategy (2)  Appropriate data collection (1)  Consideration for reflexivity (0)  Consideration of ethical issues (2)  Sufficiently rigorous data analysis (2)  Clear statement of findings (2)  Value of research (0) |
| **Potvin et al., 2019**  **Experience of Occupations among People Living with a Personality Disorder** | Specialized outpatient clinic, Canada | To explore  how occupational engagement, as experienced by people living with a Cluster B personality disorder, shapes their self-identity | N=10  Living with moderate-to-severe cluster B personality disorder (whether diagnosed or self-reported was not specified) | Mean age: 29.60 years  Aged 21-35 years  F=5 | Semi-structured interviews | Thematic content analysis | 1) When occupations are socially disapproved  2) When overinvestment/overengagement in occupations is socially disapproved  3) When occupations are socially valued. | Clear aims (2)  Appropriate qualitative method (2)  Appropriate research design (2)  Appropriate recruitment strategy (2)  Appropriate data collection (2)  Consideration for reflexivity (1)  Consideration of ethical issues (1)  Sufficiently rigorous data analysis (2)  Clear statement of findings (2)  Value of research (2) |
| **Sagan, 2017**  **The loneliness of personality disorder: a phenomenological study** | Mental health online networks, UK | To investigate the experience of loneliness amongst people who have been diagnosed with borderline personality disorder | N=7  Diagnosed with borderline personality disorder | Aged 25-61 years  F= N/A | Interviews | Thematic analysis | 1) Individual ways of thinking of and managing the experience of loneliness | Clear aims (2)  Appropriate qualitative method (2)  Appropriate research design (2)  Appropriate recruitment strategy (1)  Appropriate data collection (1)  Consideration for reflexivity (0)  Consideration of ethical issues (1)  Sufficiently rigorous data analysis (1)  Clear statement of findings (1)  Value of research (2) |
| **Sagan, 2020**  **The lonely legacy: loss and testimonial injustice in the narratives of people diagnosed with personality disorder** | Online fora and announcements at self-help groups, UK | To explore the lived experience of loneliness among a group of people diagnosed with the contested diagnosis of borderline personality disorder | N=14  Diagnosed with borderline personality disorder | Age; N/A  F= 7  Transgender= 2 | Unstructured interviews | Thematic analysis | 1) Loss  2) The experience of loneliness  3) Dissociation  4) Use of self-harm/suicidal ideation | Clear aims (2)  Appropriate qualitative method (2)  Appropriate research design (2)  Appropriate recruitment strategy (1)  Appropriate data collection (1)  Consideration for reflexivity (0)  Consideration of ethical issues (2)  Sufficiently rigorous data analysis (2)  Clear statement of findings (2)  Value of research (2) |
| **Sheffield et al., 1999**  **A qualitative examination of borderline personality disordered (BPD) patient’s interpersonal dynamics and underlying paradoxes** | Outpatients, US and Australia | To explore paradoxical dynamics evident in borderline personality disorder patient narratives of relationship events | N= 10  Diagnosed with borderline personality disorder | Age: not stated  F=9 | Relationship episode narratives | Grounded theory | 1) The intimacy dilemma: both closeness and distance are desired and intolerable  2) Self-destructiveness is self-protection | Clear aims (2)  Appropriate qualitative method (2)  Appropriate research design (2)  Appropriate recruitment strategy (1)  Appropriate data collection (2)  Consideration for reflexivity (0)  Consideration of ethical issues (0)  Sufficiently rigorous data analysis (2)  Clear statement of findings (2)  Value of research (0) |
| **Shepherd et al., 2017**  **Seeking to understand lived experiences of personal recovery in personality disorder in community and forensic settings – a qualitative methods investigation** | Community and forensic clinical settings, North of England, UK | To map out pertinent themes relating to the recovery process in personality disorder as described by individuals accessing care in either community or forensic settings | N=41  Diagnosis or self-reported personality disorder  (Most participants self-identified as having been diagnosed with an emotionally unstable personality disorder, with some also reporting a diagnosis of dissocial personality disorder) | Aged 18-60 years  F=23  White= 36  Other= 5 | Semi-structured interviews | Thematic analysis | 1) Understanding early lived experience as informing sense of self  2) Developing emotional control  3) Diagnosis as linking understanding and hope for change  4) The role of mental health services. | Clear aims (2)  Appropriate qualitative method (2)  Appropriate research design (2)  Appropriate recruitment strategy (2)  Appropriate data collection (2)  Consideration for reflexivity (0)  Consideration of ethical issues (2)  Sufficiently rigorous data analysis (2)  Clear statement of findings (2)  Value of research (2) |
| **Sorensen et al., 2019**  **Struggling to be a person: Lived experience of avoidant personality disorder** | Outpatient clinic, Norway | To better understand the subjective lived experience of persons diagnosed with avoidant personality disorder (AVPD) | N=15  Diagnosed with avoidant personality disorder | Mean age (SD): 33 years (9)  aged 20-51 years    F= 9 | Semi-structured face-to-face interviews | Interpretative phenomenological analysis | 1) Struggling to be a person  - Fear and longing  - A doubting self | Clear aims (2)  Appropriate qualitative method (2)  Appropriate research design (2)  Appropriate recruitment strategy (2)  Appropriate data collection (1)  Consideration for reflexivity (2)  Consideration of ethical issues (2)  Sufficiently rigorous data analysis (2)  Clear statement of findings (2)  Value of research (2) |
| **Smith, 2017**  **Experiences of dialectical behavioural therapy by adults diagnosed with borderline personality disorder** | Dialectical behavioural therapy programme in North west England, UK | To explore the overall experience of an NHS-delivered community-based dialectical behavioural therapy programme by individuals diagnosed with borderline personality disorder | N=6  Diagnosed with borderline personality disorder | Mean age: 26 years  Aged 22-30 years  F= 6  White= 5  White other= 1 | Semi-structured interviews | Interpretative phenomenological  Analysis | 1) Therapeutic group factors  2) Therapist factors  3) Personal change  4) Challenges to overcome  5) Personalized problem solving  6) Opposing expectation | Clear aims (2)  Appropriate qualitative method (2)  Appropriate research design (2)  Appropriate recruitment strategy (2)  Appropriate data collection (1)  Consideration for reflexivity (2)  Consideration of ethical issues (2)  Sufficiently rigorous data analysis (2)  Clear statement of findings (2)  Value of research (2) |
| **Spodenkiewicz et al., 2013**  **Living from Day to Day – Qualitative Study on Borderline Personality Disorder in Adolescence** | University psychiatric centres in France, Belgium, and Switzerland | To explore the subjective experience of adolescents with borderline personality disorder using a qualitative research method, according to two axes: childhood, and future | N= 100  n (personality disorder group) = 50  Diagnosed with borderline personality disorder | Mean age (SD): 16 years (1.1)  Aged 13-18 years  F= 88% | Interviews | Interpretative phenomenological analysis | 1) Emotional experiences  2) Interpersonal experiences  3) The representation of self  4) The structuring of discourse | Clear aims (2)  Appropriate qualitative method (2)  Appropriate research design (2)  Appropriate recruitment strategy (1)  Appropriate data collection (1)  Consideration for reflexivity (0)  Consideration of ethical issues (2)  Sufficiently rigorous data analysis (2)  Clear statement of findings (2)  Value of research (2) |
| **Vandyk et al., 2019**  **Why go to the emergency department? Perspectives from persons with borderline personality disorder** | Tertiary care hospital in eastern Ontario, Canada | To explore frequent emergency department use by persons with borderline personality disorder | N=6  Diagnosed with borderline personality disorder | Aged 22-66 years  F= 5 | Semi-structured interviews | Thematic analysis | 1) Pathways to the emergency department  2) Cyclic nature of emergency department use  3) Coping skills and strategies | Clear aims (2)  Appropriate qualitative method (2)  Appropriate research design (2)  Appropriate recruitment strategy (2)  Appropriate data collection (1)  Consideration for reflexivity (2)  Consideration of ethical issues (2)  Sufficiently rigorous data analysis (2)  Clear statement of findings (2)  Value of research (2) |
| **Vardy, 2011**  **The experience of aloneness in borderline personality disorder** | Northfield’s Clinic, University of Wollongong or at Wesley Private Hospital, Australia | To investigate the experience of time alone as described by  patients with borderline personality disorder | N=12  Diagnosis of borderline personality disorder | Mean age: 36.3 years  Aged 19-56 years  F=12 | Interviews | Phenomenological analysis | 1) General Feeling about Time Alone  2) The Essence of a Negative Experience of Time Alone  3) The Essence of a Positive Experience of Time Alone  4) Both a Negative Experience Alone and with Others | Clear aims (2)  Appropriate qualitative method (2)  Appropriate research design (2)  Appropriate recruitment strategy (2)  Appropriate data collection (1)  Consideration for reflexivity (1)  Consideration of ethical issues (2)  Sufficiently rigorous data analysis (2)  Clear statement of findings (2)  Value of research (2) |
| **Williams, 2016**  **What Goes Unheard When Struggling Mothers Speak? A Qualitative Study Using the Adult Attachment Interview.** | Psychotherapy and Complex Needs Service, NHS, London, UK | To give voice to mothers who have been silenced in many ways, to learn from their struggles and to help services develop more effective ways to reach ‘hard to reach’ vulnerable mothers | N=8  Personality dysfunction that would meet criteria for diagnosable personality disorder assessed via International Personality Disorder Examination | Aged 20-50 years    F= 8  White British= 7 | Adult attachment interviews | Thematic analysis | 1) Love and loss  2) Change and Confusion  3) Families and normality  4) Safety and boundaries  5) Strength and vulnerability | Clear aims (2)  Appropriate qualitative method (2)  Appropriate research design (2)  Appropriate recruitment strategy (2)  Appropriate data collection (2)  Consideration for reflexivity (2)  Consideration of ethical issues (2)  Sufficiently rigorous data analysis (2)  Clear statement of findings (2)  Value of research (2) |

^^[[1]](#footnote-1)^^ CASP (critical appraisal scoring program): a score of 2 denotes that criterion is fully met, a score of 1 denotes that criterion is partially met, a score of 0 denotes that criterion is not met

SD: Standard deviation

F: female participants

n: number of participants

BPD: Borderline personality disorder (also known as emotionally unstable personality disorder; EUPD)

1. [↑](#footnote-ref-1)
