# Supplementary material for: The experience of loneliness among people with a “personality disorder” diagnosis or traits: a qualitative meta-synthesis
Source: BMC Psychiatry. 2022 Feb 17;22:130. doi: 10.1186/s12888-022-03767-9 (PMC8855579; doi:10.1186/s12888-022-03767-9)
Supplement: Supplementary file 2 — Additional file 2. [file 12888_2022_3767_MOESM2_ESM.docx]

Supplementary Table 2. *Themes, subthemes and illustrative quotes by authors and participants contributing to meta-synthesized themes*

| Meta-synthesized themes | Themes, sub-themes and quotes from authors contributing to the meta-synthesis | Illustrative quotes from or interview scripts of participants contributing to the meta-syntehsis | Source |
| --- | --- | --- | --- |
| **Theme 1:**  Disconnection: A “haunting alienation”:  **This theme reflected an overwhelming sense of loneliness characterized by feelings of inevitable alienation, disconnection, and otherness/differentness from society at large. These experiences were often described in relation to persistently feeling misunderstood, out of place, inadequate, invisible, and/or dead.** | 1.Being in the world: Estrangement, inadequacy and despair:  2. Therapeutic group factors:  - Participants’ interview excerpts indicate that, prior to DBT, they had lived a number of years alone with BPD, a life of being misunderstood.  3. When Occupations Are Socially Valued:  4.Relational experiences:  - The feeling of loneliness and solitude among adolescents with BPD was a recurrent theme when they talked about their day-to-day interpersonal relationships.  - They tended to compare themselves to their peers, and these comparisons were very often to their disadvantages.  5. Struggling to be a person: - Lonesome struggles in which their (participants) everyday experiences were simultaneously scrutinized and shunned.  - They had little sense of belonging to a social group or feelings of bonding or intimacy; they also displayed reduced knowledge of co‐operative and normative behaviors.  - Most often, they (participants) concluded that the other person did not understand them (or) failed to take them seriously.  - Some participants described their longing for connection as something related to a sense of being known by other people.  - This felt state of aloneness almost seemed a preconceived condition of life: it is just the way it is and always has been. Further, their aloneness appeared colored by a lack of hope, even despair, at ever managing to change their condition  6. Feeling flawed, insecure and fearing rejection:  - Many participants expressed feeling insecure and like they did not belong around others. They reported worrying that they would say or do the wrong thing around others, and described feeling like an outsider.   - When she’s consumed by her thoughts and her misery she can feel alone in a room full of people   7. Individual ways of thinking of and managing the experience of loneliness:  - The words used to describe the feeling of loneliness, its “haunting alienation” and not fitting in anywhere.  - This inability, an impossibility to “connect, like other people do” was spoken of repeatedly.  8. Exclusivity within rituals: negative transient emotions reinforcing low EE:  9. The external world:  10. Love and loss:  - The participant’s described their need to feel they belonged somewhere, however often reported feeling confused about where they belonged or lacked any sense of belonging altogether.  11.Narratives of loneliness:  12. We are one (but not together):  - Tabitha’s quotation seemingly demonstrated a sense of felt rejection from the group as she didn’t “fit in”. | *“I had a lot of friends, but I never felt a part of the group”*  *“I felt… separated in a way, not quite in there with the rest”*  *“it’s like especially with borderlines, we feel like we’re being misunderstood all the time by everyone”*  *“I felt like I was out of place. I did not feel competent at all. I just felt like an impostor, that I should not be there”*  *“They have everything and I have nothing.”*  *“I wish I could open up and show (what is inside), but I do not know exactly how to do it”*  *“Nobody knows me, and I have never felt seen.”*  *“There comes heaviness, like ‘now you are alone again little man, and you will never manage this; you will die alone’”*  *“I feel that I’m different to everybody else like I’m an alien. Like I’m not right. They’re right and I’m wrong and my opinions don’t matter because they’re right and I’m not able to make really good decisions.”*  *“I just never felt like I fitted in anywhere. It was that sort of thing that you were there but you were watching from the outside all the time. I was always, always out, going to parties, going to clubs, going to live music and doing all these things but I always felt uncomfortable. I always felt uncomfortable. It is not that I sit on my own all the time but I do feel this huge emptiness and that’s another part that’s always gone on in my life.”*  *“I just think I’ve been lonely all my life. Even though there were times when there were people around me if you like, who I thought were my friends, I don’t think I ever really, I’ve ever felt part of anything.”*  *“it’s fearful, overwhelming loneliness […] like I’m a freak of nature that should never have been born and therefore this is kind of the – this is just – what my life is, what’s, what I was destined for. You know, this is almost my punishment for surviving the rest of it.”*  *“That’s all I would do […] It was to block out the world, completely block out the world. I think it was because I felt so disconnected from the world; the world didn’t feel particularly real.”*  *“I’m married and have two boys and a granddaughter, who is beautiful […] but a lot of the time, most of the time […] I don’t feel the connection. I’m alone and lonely and I would do anything not to be like this. I don’t know how to feel real – although I pretend to be real and maybe if I pretend long enough perhaps I’ll be real.”*  *“But it doesn’t work like that […] sometimes the loneliness gets so bad […] that you think – I think I’m already dead. As if I’m already empty, will I be like this when I’m dead?”*  *“I’ve tried to join in more, I’ve tried to give more feedback if you like. But...I still don’t feel a part of the group. I feel like there’s everybody else and me. So...that gets me down, that I’m not a part of it.”*  *“… if you imagine floating through life and not really being able to connect with anyone at all because you have to hide yourself, you have to put on a mask to show the world, that you’re not mentally ill because it, there’s such a stigma behind it”*  *“loneliness is not feeling.. . is feeling I’m not part of anything. I’m not part of the world. I just feel like I’m completely shut off and that nobody, nobody notices, nobody cares, nobody – I’m not good enough. Yes, definitely I get that feeling I’m not good enough to be part of society.. .when you have that deep-rooted, when you can actually feel it here the loneliness and the self-hatred, you can’t physically get out of that and it is with you all the time”*  *“It didn’t matter what I did. It felt as if it was always for somebody else and not for me. That somewhere I’d got lost and all I could do was react and be what other people wanted me to be and that made me feel incredibly lonely at that early stage”*  *“I used to self-harm because that made me feel real in that moment.. .sometimes the loneliness gets so bad.. .that you think - I think I’m already dead”*  *“And then because I felt like I didn’t fit in with the group in certain ways or like they didn’t really get where I was coming from with that stuff. They make me feel even more like they have an outsider”* | Miller, 1994  Smith, 2017  Potvin et al, 2019  Spodenkiewicz et al., 2013  Sorenson et al., 2019  Vardy, 2011  Sagan, 2017  Clarke and Waring, 2018  Gillard et al., 2015  Williams, 2016  Sagan, 2020  Dyson & Brown, 2016 |
| **Theme 2:**  Alienation arising from childhood experiences  **This theme illustrated that the sense of disconnection and alienation experienced appeared to originate from early alienating and rejecting experiences within the family unit.** | 1. Pre MBT:  - Prior to therapy, LERs (lived experience researchers) felt they were chronically stuck in very rigid forms of relating.  - Examples of this might include being stuck either as a “black sheep” in a family.  2.Mood states that impacted the self:  - This focus on disconnection from others was a common theme, along similar lines as the inhibition that is associated with avoidance.  3. Understanding early lived experience as informing sense of self:  - For other participants, early life experience was characterised by a sense of alienation from their family, lead-ing to them struggling to develop a sense of their own ‘place’ within the social unit.  4.Struggling to be a person:  5.Feeling flawed, insecure and fearing rejection:  - She felt alone and used when her parents singled her out from her seven other siblings to do things for them but then subsequently ignore her afterwards.  6. Love and loss:  - Psychological separations were present in their narratives also whereby participants frequently described feeling emotionally distant from their parents, or as though they were kept at a distance from their parent/s  - A further sense of rejection came from feeling excluded from dyadic family relationships, often involving two parents or a sibling and parent  - Feeling in the way, rejected, and ostracized by family members had caused many of the participants to feel abandoned as children  Theme: Families and normality:  - Feeling like a disappointment to parents and regarded as the ‘black sheep’ was not uncommon and many of the participants described feeling that their sibling was favored and given preferential treatment.  7.Dangerous and Troubled Relationships, Isolation, and Little Support: | *“it was a constant state of panic as though everyone was going to get me. It was feeling excluded all the time.”*  *“My parents gave me all the freedom I wanted, but they were so busy with their own lives. I was all alone. I lived in a house but not a home.”*  *“I had a lot of depression and down days, when I think back now just not fitting in even the foods that I liked were totally different I had nothing in common with the family that I lived with and brought up with. Not in the food, nothing.”*  *“Nobody knows me, and I have never felt seen. Not even my mother knew me like that. I know I have missed it. I never felt loved”*  *“I never felt like I belonged at home, never felt it. I was treated totally different, I could be a kid at my auntie’s but I couldn’t be a kid at home, I had to be quiet and that’s not good for a kid”*  *“[mum] was always distracted by something else so I was sort of… I always felt like she was there and along her list of things, I was sort of there… there was always that sort of distance and I couldn’t quite get close enough to have a relationship with her because she would put a lot of other stuff in the way”*  *“I wanted a bit more out of her, like I knew there was something, lack of her inside … when I was younger I knew I was missing something but I couldn’t quite understand what the hell it was at that age”*  *“My mum was there for us and loved us and all that but on the emotional side, she wasn’t there for our emotional needs and nor was my dad.”*  *“I’m the black sheep really, they are more there for each other because they are real siblings, I am only a half, and two’s company three’s a crowd and that’s just how I feel”*  *“I have no family, I didn’t have anything in my life . . . My head was f***** up since I was four, I can remember my mum telling me she hated me.”* | Johnson et al., 2016  Narayanan and Rao, 2018  Shepherd et al., 2017  Sorenson et al., 2019  Vardy, 2011  Williams, 2016  Gilbert et al., 2013 |
| **Theme 3:**  A thwarted desire for closeness and connection:  **This theme illustrated how people with “personality disorder” diagnoses/traits described a hindered desire for closeness, connections, and real relationships that seemed to arise from unmet social needs.** | 1. Having supportive relationships is important:  - All participants described how vital close relationships, such as family and friends.  2. Desire for emotional containment:  - Our accounts described a desire or perceived need for more consistent support outside the group (therapy).  3. Pre MBT:  - The result of these limited social contacts was that LERs felt very alone, unable to connect with  other people emotionally or make sense of their intentions.  4. Mood states that impacted the self:  - (participant) suggest a need for a relational self in accordance with the Indian context.  5. Struggling to be a person:  The superordinate theme of “struggling to be a person” reflects the participants’ ongoing efforts to constitute themselves as functioning persons among others. This struggle seemed to be related to trying to emerge as relational individuals within an experienced life world of isolation.  Theme: Fear and longing:  - What came across in the descriptions of all of the participants was a longing to connect and thus belong to the fellowship of others.  - Furthermore, however, close the participants felt to their animals, a longing for human contact still seemed to linger in their efforts to participate in social contexts.  6. Solitude, Fearing Relations–Longing for Love and Fellowship:  7. Personal goals and/or achievements during recovery:  - (Participants would like to) be able to talk about their feelings and allow themselves to feel vulnerable in close relationships, tolerate fears of rejection and abandonment | *“When you was by yourself, and you got no partner or anything, do you know what I mean – it’s a bit depressing […] I ain’t got a girlfriend or anything.”*  *“I struggled … my anxiety levels increased, increased, increased. And it wasn’t made particularly clear to me that I could also access the team outside of the group situation”*  *“I would like to interact better with people, be more forthcoming, more sociable, more gregarious, less paranoid”*  *“I want to have relationships with people but when I feel down it doesn’t matter, I don’t take contact because I’m about to die anyway”*  *“I wish there was someone to hold my hand supporting me over the obstacles.”* | Musa ,2019  Bradley-Scott, 2017  Johnson et al., 2016  Narayanan, 2018  Sorenson, 2019  Perseius et al., 2005  Katsakou et al., 2012 |
| **Theme 4:**  Paradox: Push for both Closeness and Distance:  **This theme is characterized by a simultaneous urge for closeness and distance. In this paradox, people with “personality disorder” diagnoses/traits described feelings of loneliness and emptiness that triggered a need for intimacy, however fears and perceptions regarding the social world countered these urges. People with “personality disorder” diagnoses/traits employed a variety of approaches to cope with their fears of the relational world, such as hiding and masking insecurities.** | 1.The intimacy dilemma: Both closeness and distance are desired and intolerable:  - Wishes and behaviors which reflected seeking both closeness and distance within the same situation, as well as across situations.  - Subjects typically manifested the dilemma in a way that made it impossible to resolve, and in many instances managed to simultaneously "pull" for both closeness and distance.  - A patient wants "good communication relationship," but is afraid that any relationship will "become abusive at some point." So, when a man whom she has dated for 3 years proposes marriage, she ends the relationship because she is "too afraid," even though he is "very kind" and "patient."  - Closeness was consistently undermined deliberately, sometimes as a way of self-sabotaging in order to punish self, but more often as a way to protect self from the possibility of being abused, controlled, or abandoned.  - At the same time, closeness was greatly desired, while distance was associated with loneliness  2.Fear of intimacy versus intrusion:  -In this theme, the women reported confusion and a paradox in the struggle between their longing for intimacy and their fear of intimacy and self-harm, where the latter can be a way of protecting oneself  -The women’s longing for intimacy was a paradox, as they  were frightened of both mental and physical intrusion, especially by men.  3.Fear and longing  - All of the participants conveyed a sense of being locked in a conflicting stance of fear and longing in their relations to both themselves and others.  -They felt a desire to connect with others but, at the same time, feared becoming close. They both longed for solitude and feared aloneness.  - “The other” was viewed with great suspicion by all of the participants  - All of the participants described how their efforts toward (and inferred longing for) connection was accompanied by a simultaneous fear of others’ possible opinions, motives, or agendas.  -They (participants) described what can be characterized as an ambivalence of efforts: reaching out for relationships yet, at the same time, withdrawing from them. Within this inhibiting state, the participants seemed to feel increasingly vulnerable as they grew closer to another person, fearing what might happen to them if they were to be exposed.  4.Solitude, Fearing Relations–Longing for Love and Fellowship:  -There is also ambivalence between the wish for  love and fellowship and the self-hate that tells patients that they are not worthy to be loved.  5. We are one (but not together):  - Indeed, this negotiation between connection and disconnection may possibly be the felt experience of their own attachment processes.  - Several participants spoke about their “insecure attachment” which itself implies a felt push-pull between connecting with another, and fear of losing oneself in the other  Fear and longing:  -All of the participants conveyed a sense of being locked in a conflicting stance of fear and longing in their relations to both themselves and others. They felt a desire to connect with others but, at the same time, feared becoming close. They both longed for solitude and feared aloneness. | *“I want somebody to love me and want me and care. There's nobody there because I don't want them.”*  *“I long for someone to love me, but I realize I am difﬁcult to understand. When I am suffering, I cannot bear being with anyone. In this painful state, I do not answer the phone or the doorbell. I do not want anyone to visit me. If only somebody would come before I become so destructive that the only thing left to do is to scald myself with boiling water to obtain relief from this pain.”*  *“Don’t touch me! I don’t like anybody and nobody are allowed to like me. Do you hear me, but the little girl inside is still striving to be loved”* | Sheffield et al., 1999  Holm & Severinsson., 2010  Sorenson, 2019  Perseius et al., 2005  Dyson & Brown, 2016 |
| Subtheme 4.1  A rejecting and hostile external world:  **Despite the desire for fellowship, people with “personality disorder” diagnoses/traits described a perception of a hostile and rejecting world that fed into the urge to psychologically withdraw and retreat from the social world to maintain a sense of safety. This fear of rejection seemed to arise from early experiences; and are further exacerbated by turbulent relationships and trauma.** | 1.Solitude, Fearing Relations–Longing for Love and Fellowship:  - The fear of being rejected puts them (participants) in a position of the strong solitaire or testing if another person really can be trusted  2. Clinging is distancing:  - Test for possible closeness  3. Stigma  - Negative attitudes of professionals could increase feelings of rejection and abandonment in patients with BPD  4. Coping strategies  - They (participants) did not want to burden anyone (and) feared rejection  - (Patients would ask themselves in social interaction) would they be accepted or rejected?  5. Fear of intimacy versus intrusion:  - The women reported that their emotional pain increased when they were alone and abandoned.  - It is understandable that the women are fearful as a result of traumatic experiences  6. Needs:  -Attachment and abandonment experiences from childhood were raised by several participants.  -Participants with these experiences expressed how they would often push away relationships through fear of rejection.  7. Fear and longing:  -They (participants) expressed that these solitary periods felt like their best option for gaining both freedom and restitution from the perceived impossible demands of others—and a corresponding fear of rejection.  - Some participants expressed a feeling of being treated unfairly by others who seemed to always have their way.  As they fearfully acquiesced to the assumed wills of others, frustration and anger would build up within them.  Most of the participants described how they stayed vigilant when with others; they were always on guard against possible signs of danger in their social surroundings.  -They described how they needed to protect themselves from exposure  8. The essence of a positive experience of time alone:  - They (participants) feared being rejected and hurt  Relating is effortful and self-sacrificing:  - Participants found it took a lot of effort to relate, to please others and to communicate.  -Participants described times when they sought time alone because being around others invaded their senses and overwhelmed them. Participants reported feeling attacked, controlled and that their boundaries had been intruded upon.  9. Loss and love:  -Related to both love and loss were the repeated experiences of feeling rejected; participants frequently described feeling dismissed, unsupported and not understood by their caregivers  10. Seeing the world differently due to MBT: a positive shift in experience:  - Supportive and positive interactions with group members assisted Kevin to see that not everyone is “void, callous and cold”, which he had previously believed following an abusive past.  Experiencing group MBT as unpredictable and challenging:  - Sarah (participant) spent much of her time expecting to be judged by other group members.  - John (participant) experienced intense anxiety prior to each group session due to fears about how the group would react to him, although he acknowledged that these fears had not been realized  11. Self-Image Problems  12.Understanding early lived experience as informing  sense of self:  -Within the context provided by their social networks, participants saw some elements of behaviour as constituting a destructive aspect of themselves.  13. Distance between us:  - Participants described how they at times wanted to keep the self hidden from others for fear of being judged negatively, being hurt, or abused.  - All participants spoke about times when they attempted to hide the physical and psychological self from others.  14. Individual ways of thinking of and managing the experience of loneliness:  15. The lived experience of personality disorder  -Interviewees reported feeling alienated by a hostile outside world and needing to isolate themselves within their internal world in order to feel safe, often at the cost of a punitive or harmful relationship with the self.  The external world:  - Interviewees spoke of a sense of failing in the outside world as a result of the difficulties and struggles they experienced with fitting in  16. Moving from distrust and defensiveness to opening up to others  - They (participants) explained that they were reluctant to talk about difficult issues, for fear of feeling exposed (and) vulnerable. | *“To trust others I have to test the relationship very much, and who ever can cope with that in the long run?”*  *“If he doesn't run, then he might be able to take care of my needs.”*  *“I was admitted to a psychiatric hospital for some weeks and the worst thing during my ﬁrst days was the fear of being abandoned by the nurse I trusted.” “When she left the room, I thought she would never return again and I trembled with fear, wondering where she was going, feeling abandoned and alone. The only thing left was to cut myself to obtain relief.”*  *“Well, like in relationships, messing relationships up, horribly; walking away from people who care about me; cutting people out of my life…”*  *“I have a temper now, even if I do not show it. It is a bit creepy, and it hurts. It has to do with how I always bend my neck for others, and I cannot be bothered anymore”*  *“I get quiet. I do not dare say much. I just sit and attend to whatever goes on”*  *“I just felt like I was all over the place and I had all of these funny, all these demands put on me. Like all these demands”*  *“Well I suppose it’s just that - you just feel like all your senses are invaded. Every single sense is invaded. Your nerves are just … just underneath the skin”*  *“Oh my stomach starts to churn. I get a sensation of sort of moving … moving away even if I’m not physically moving away. I have a sensation that I’m moving away [I: yeah] and as I said I drink to comfort myself [I: yeah] and to relax more so what they were saying didn’t bother me so much or what they were doing didn’t bother me so much [I: yeah] or but now you see the DBT I’d leave [I: ah, ah] you see where I know that, that choice is there.”*  *“Relations are some of the must important things in life but sometimes some of the most difficult things.”*  *“It (relations) turns into different sorts of conflicts, troubles. Relations are some of the things that are difficult in life.”*  *“I won’t let many people in, I choose my circles… who I speak to even smaller… I still choose not to speak to a lot of people about it.”*  *“I very much keep people at arm’s length. So then people stop trying to engage with me. So some of it is self-imposed but I don’t necessarily do it on a conscious level. It’s a form of protection I guess. […] when times are tough I go inside myself and I retreat from the world and I hide in my house and this is where the loneliness comes in I suppose.”*  *“My perception of people involved in my care undergoes like a paradigm shift. The people who I once thought were caring and supportive become … tyrants who are trying to manipulate me and erode my freedom … human beings are all evil and corrupt and everyone’s out to take advantage of me …”*  *“At the moment, someone asks what have you done with your life, you know, I have done absolutely nothing, I left school at twelve, drink, drugs, prison, come out, a locked ward, that’s it. I can’t say that to somebody that’s been to university, has got a good job … I said once in an AA meeting about being in prison and someone went ‘argh, that’s disgusting’, and that f****** kicked me in the teeth.”*  *“Like in relationship with my mum or my dad, being able to express anger from the past… it’s more of a fear of losing control and thinking “have I let people down? and they are not gonna care for me and then how am I gonna cope?”* | Perseius et al., 2005  Sheffield et al, 1999  Juurlink et al., 2019  Miller, 1994  Holm & Severinsson., 2010  Lamph, 2018  Sorenson, 2019  Vardy, 2011  Williams, 2016  Lonargáin & Hodge, 2017  Falklöf & Haglund, 2010  Shepherd et al., 2017  Agnew, 2016  Sagan, 2017  Gillard et al., 2015  Katsakou, 2016 |
| Subtheme 4.2:  Ways of managing the paradox:  **People with “personality disorder” diagnoses/traits described a wide range of methods to cope with their urge to connect and counter feelings of loneliness yet avoid corresponding perceived demands and fears associated with the social/relational world.** | 1.Coping strategies:  - Most restricted their relationships to ones in which they felt in control such as occupational roles or volunteer positions  2. Needs:  -Participants with these experiences expressed how they would often push away relationships through fear of rejection.  - Just under half of the participants described themselves as socially withdrawn.  - One participant described a fear of others not liking him, which had resulted in him developing a guarded approach to others, as a method of self-protection.  3. When Occupations Are Socially Valued:  - Some participants strive to ﬁnd ways to be in contact with others, while keeping them at a distance  Theme: When Overinvestment in an Occupation Is Disapproved:  - Three participants described that they escaped a diﬃcult  reality by living a virtual life by means of social networks and digital technologies, at the expense of investing in their “real” social networks. In addition, keeping a virtual distance assisted participants in maintaining connections and relationships with others, without feeling overwhelmed  4. Protection and caring:  5. Cyclic nature of emergency department use:  -The participants explained how feelings of loneliness  or experiences of loss perpetuated a state of mental deterioration that ultimately led to an ED (emergency department) visit.  - ED were prompted by… intense emotions such as loneliness and negative self-perception often triggered by loss.  6. Managing and finding respite:  7. Fear and Longing:  - As the participants did not feel “normal” as they observed others to be, it became crucial that they acted “as if—  normal” whereas simultaneously hiding their perceived shortcomings.  - Participants used words such as “putting on a mask” and “following the templates of behaving,” as opposed to “the real me” or “the sick me.”  - The participants described attempting to act as if they were happy, content, strong, or competent.  -Relating to others was also described by some participants as easier if the interaction was focused on a common activity with organized guidelines for behavior, such as playing sports.  -Those who had small children or animals described these connections as important, genuine, and true. These relationships evoked tender feelings in the participants and gave meaning to life. Their own sense of vulnerability seemed to become somewhat forgotten in the moment of providing care and protection  -Some (participants) chose routes for walking that were less populated, or they interacted with others mainly through their computers or text messages.  -Some (participants) strove for perfect behavior, as doing so could conceal their perceived flaws or possible defects. Others attempted to become almost invisible; if they managed to do so, they reasoned, the likelihood of others including them in a social interaction was reduced.  8. Individual ways of thinking of and managing the experience of loneliness:  -Participants described efforts made to find anchor points, such as work, volunteering, arts activities and religion to provide some sense of connection.  9. The Mask of Normality:  - The mask of normality is also narrated as some kind of invocation—representing a will and a struggle to be normal | *“And I’m suspecting now that perhaps I sabotage my relationships as a way of avoiding being let down by people, because if I can push them away first then they can’t let me down and hurt me”*  *“Socializing is diﬃcult for me (…) I now know why I lose all my relationships. When I volunteer, it’s less present. It’s not even present at all. When I volunteer, I meet a lot of people that I do not know”*  *“It takes a long time for me to trust someone, animals I trust them straight away”*  *“I called 911 and talked to somebody, I wanted to talk to somebody or the ED: I think that’s why I am going to the hospital. It is because I’m living alone, because I get really lonely at night’.”*  *“I started going more [to the ED] after I stopped talking to my ex because, despite how bad things were, he was my main support for four years. He was like my best friend. We were really close”*  *“I ain’t got a girlfriend or anything. I hadn’t for a while, so, do you know what I mean? Makes you a bit lonely, but that’s why – you know what I mean? I compensate. And I have four dogs, they’re like my babies.”*  *“I notice that you spend incredible amounts of energy. You just spend your entire consciousness in just not … trying not to make a fool out of yourself and appear normal”*  *“if I act normal I may be regarded as normal and then I may become normal.”* | Miller, 1994  Lamph, 2018  Potvin et al., 2019  Williams, 2016  Vandyk, 2019  Musa ,2019  Sorenson, 2019  Sagan, 2017  Perseius et al., 2005 |
| **Theme 5:**  Experiences of a meaningless and empty existence:  **This theme described experiences of feeling and being psychologically and physically cut-off and disengaged from the relational world to the point of utter hopelessness and overwhelming loneliness. This theme also captured accounts of unstable lifestyles and a lack of leisure pursuits, goals or plans.** | 1.Relational experiences:  - Unlike the adolescents in the control group, the absence of mention of leisure activities in the BPD group is striking. This could increase the feeling of loneliness of these adolescents.  -It can be noted that the parents were more frequently mentioned than peers. When they did mention peers, it was to talk about their dissatisfaction.  2.The essence of a negative experience of time alone:  - All participants described instances of feeling very depressed when alone and at these times they would isolate and withdraw. They described feeling rejected, unloved, helpless and hopeless and reported becoming utterly despondent.  - Life felt meaningless at these times and individuals felt overcome with feelings of loneliness and worthlessness.  -Her loneliness is coupled with feeling gutted and feeling pathetic that she hasn’t achieved anything and doesn’t want to (from interview scripts)  *-* She thinks maybe it’s partly that she lost three close friends in a year and a half and didn’t think she’d be alive today, so she’s got no plans or goals (from interview scripts).  - Also her loneliness is related to relocating to a new place where she only knows two people (from interview scripts).    3. Individual ways of thinking of and managing the experience of loneliness:  4.Practical achievements and employment:  - They (participants) also wanted to work towards finding a job and making progress in their career, as this makes them feel more competent and ‘normal’  5. Love and loss:  - The distance created either by geography or significant age gaps were frequently referred to and appeared to present a barrier to maintaining connected relationships.  -Participants often spoke about their sibings having their ‘own lives’ and felt separate from these  6. Consequences of long admissions on daily life:  - For those living on their own, being discharged from hospital increased their sense of being isolated at home | *“In my class, I hate to see them laugh. They have everything and I have nothing.”*  I: On the couch? And what do you do on the couch?  *BV: I just watch time go by.*  I: Yeah so like nothing? Do you know what’s going – is there anything going through your mind?  *BV: That I should be doing something that this is a waste of time and – but then its like “blow it the couch is comfortable.”*  I: So can you – what are you’re thoughts on why it’s so hard to … to do something, you know when that feeling, what is that feeling do you reckon?  *BV: I guess in a way it’s loneliness that I don’t have something planned or a bit of frustration that I can’t get myself going to do something.*  *“Yeah it gets worse. It gets to the point where I’m thinking “oh I’m useless”,*  *“I’m a nobody”, and none of my friends have phoned me to see how I am, so obviously no one cares.”*  *“Loneliness […] it feels like you have lost part of yourself. Being alone […] loneliness is soul destroying. I’ve learned to cut off my emotion quite entirely – at the end of the day I don’t want to be alone but I feel I always will be and that is something I am going to have to come to terms with”*  *“I still haven’t managed to get back to work and I can’t see , I’ve been cut off because I’ve stopped working”*  *“I would go off on my own, there was no one I could trust to turn to it was like I was bullied all the way through primary school and I was frightened all the way through primary school but there was no one I could do anything with because Bruce had died by this point. I was probably about 8 or 9.”*  *“I just had friends who I knew for a few years and then moved on again… it wasn’t nice but you just have to get on with it … we moved around a lot”*  *“It was quite a shock when I went home after being on the ward for 4 months, living alone: as much as I like peace and quiet, it’s deathly quiet and weird after the ward: I underestimated how lonely and quiet it was going home”* | Spodenkiewicz et al., 2013  Vardy, 2011  Sagan, 2017  Katsakou et al., 2012  Williams, 2016  Birken & Harper, 2017 |
| **Theme 6:**  Recovery, embedded in a social world:  **This theme described the importance of finding supportive and understanding relations in one’s recovery journey and wellbeing. For some people, a sense of belonging and unity in a group therapeutic setting appeared to mitigate loneliness and the sense of otherness experienced by people with diagnoses/traits of “personality disorder”.** | 1. Building trust: a gradual but necessary process during MBT:  -Being in a group with people with similar difficulties was described as a “dream come true” by one participant and was also a key factor in helping other participants build trust.  -Through MBT he learned to give himself space before acting which enabled him to develop a range of explanations outside his immediate assumptions and thus see the world in a more balanced way.  2. A sense of belonging and community:  - Where decision-making was shared, bonds of friendship were made, where there was fun, where shared realities were negotiated and where there were experiences of uniting in a common purpose  - Voicing their newly developed sense of healthy attachment, participants began to regain, or gain for the first time, a sense of home and family:  3. Managing and finding respite:  - The support provided by a network of relationships allowed participants to garner emotional strength dealing with their PLEs (psychotic like experiences (in people with BPD)) and to feel less alone  4. Conditions of change:  - For many participants, change was said to be relational inasmuch as it was achieved with the assistance and support of others. Our participants commented on how important their relationships with others were in their recovery journey  - The most significant relationships that supported recovery were reported to be with friends, family or intimate partners or with clinicians or peers in therapy.  5. Shared understanding:  - Tilly (participant) spoke about a sense of finally being understood by others, and no longer feeling alone with the ‘condition’ of BPD.  - This understanding appeared to not only remove the loneliness of living with the painful emotions and often destructive behaviours associated with BPD, which are often misunderstood by others, but also add a sense of cohesion and relatedness among the skills group members.  6. Relationships:  - While DBT (dialectical based therapy) certainly has not freed them of familial conflict, it has given them the ability to share more with their family while caring more about themselves.  Skills training:  clients indicated a preference for group over individual skills training. The group provides support from other clients struggling with similar life issues.  7. Self within others:  - There was a sense of belonging and relief through the shared understanding of ‘BPD’ difficulties.  Theme: Self in relation to others:  -Most people noted increase perspective taking in relationships and thinking of themselves as separate but connected to others  8. At the point of the timelines:  -Small social contacts were enjoyed so much more than previous attempts at socializing through work roles or in families.  -LERs (lived experience researchers) directly contrasted this type of engaged feeling with their constantly feeling empty and irrelevant prior to MBT. The most important aspect of that connected feeling through mentalizing was a sense of a social and moral life-purpose  9. Confronting interpersonal difficulties and practicing new ways of relating:  - They (participants) began to perceive the group as a nurturing environment, where they felt understood and supported, this made them feel less isolated and more ‘normal’  10. Personal goals and/or achievements during recovery:  - Participants explained that they would like to improve their relationships, socialise more, be less isolated, build more supportive relationships in their lives, and end unsupportive or abusive relationships  11. Dimensions Related to the Environment:  - Ten participants said they maintained or hoped to maintain healthier and stronger relationships.  - Eight mentioned that this meant to create or enlarge their social network and/or minimize conﬂictual relationships.  12. Diagnosis as linking understanding and hope for change:  13. Evaluation of therapy:  - Being able to identify with others became important therapeutically as it provided participants with a renewed sense of identity, effectively reducing their level of experienced loneliness and questioning of the self, allowing them to lose some of the self-stigma they had attached to the diagnosis of BPD, and develop the courage and confidence to socialise again  14. Empowerment through inclusion: - When Andrea used a self-harming behaviour, the clients worked together to extend support and a sense of belonging to her, drawing her back into the community and adhering to the TC (therapeutic community) value of client member safety.  - The clients tell Andrea they are worried for her because they care. Andrea leans over the arm of the sofa and bursts into tears. Erica passes her tissues. Andrea says she struggles to hear that because no one has ever cared about her.  15. Transforming negative transient emotions in to high EE (emotional energy):  -Tessa states EMs (emergency meetings) are a really good way to feel included in the community. They explain when you are new, being part of an EM and helping someone through a crisis can really make you feel part of the therapeutic community.  16. Recovering or discovering the self – reconciling the internal and external worlds:  -Much of this specific understanding of recovery was about finding ways of being – of thinking, feeling and acting – that enabled them to live in the outside world: | *“It’s all about human contact. I think a lot of people here realise what it’s like to be lonely, we all know what it’s like so we all make an extra effort to be friendly.”*  *“It’s like having a family all under one roof.”*  *“It’s the family I never had.”*  *“I went into wellbeing groups as well and society groups […] it was nice to understand that it’s not just me making all these things appear in my head like it is a thing where they come back”*  *“we are so deeply connected” and they “make me feel like I’m not alone, there’s more people in there, in this world with my same feelings, and we can, we can get better.”*  *“I’ve pretty much spent my entire life being surrounded by people who don’t understand. Now I’m in a room with other people who, like, I’ll say something and they’ll go “yes, I do that,”……. it’s good just knowing that I’m not the only one…….talking to the other girls in the group, I know that when they say “I understand”, there’s a very good chance that they actually do.”*  *“finally meet a group of people that no matter how weird whatever you’re going to say is they’ll be like “yeah, I get that as well”. And it’s like, just the fact that you’re not alone anymore….”*  *“I can talk to my family more effectively about what's going on in my life, whereas before I was afraid to tell them what was happening. They never knew what was going on in my life, they never knew when I was having a hard time. I can talk to my kids better about it .... And my friends, it's just easier to talk to them about what's going on.”*  *"When we got together before, I was very passive, and now I have the skills, we can talk about anything. I no longer have taboo subjects . . ."*  *"I can go on vacation or go on an outing and enjoy it, not be angry at the way every single person in the place looked at me or . . . bumped into me."*  *“to finally know that there are other people … I’m not a freak, that it actually has a name … gave me a relief that there was help out there.”*  *“it’s sort of a sense of relief that “God, it’s not just me. It’s not just me that feels this way about things, or has problems”*  *“Those weeks when I couldn't meet up with care coordinator, I felt a little bit lonely and confused, frustrated, stuff like that. But, generally speaking the group therapy, it was good actually.”*  *“I’ve always thought that it was me, everything was to do with me. And now I’ve started to look outside a bit more. Instead of inside”*  *“it is very comforting to be with a bunch of people who know where I’am coming from, so I don’t feel like a weirdo… it’s almost nurturing for me… this is almost like an adopted family for me… I can actually feel myself doing all the learning that perhaps I should have been doing donkey years ago”*  *“I had no friends, I had nothing. I made them run away. Now I have plenty and it’s fun.”*  *“I started going to a hearing voices group, which was near where I lived, so that made things a lot easier knowing that I was with like-minded people.”*  *“It was good knowing that there was other people out there… it got rid of that kind of loneliness and am I a freak?”*  *“It was comforting knowing that there’s people out there that have issues like me… it just makes you feel normal, it makes you feel like you’re not that freak you thought you were, that you’re a normal human being and you just have problems.”*  *“For me personally it means … sort of reintegration into the community and sort of mainstream society … to combat the feeling of alienation that I experience.”* | Lonargáin & Hodge, 2017  Castillo et al., 2013  Musa, 2019  Donald et al., 2017  Smith, 2017  Cunningham et al., 2004  Bradley-Scott, 2017  Johnson et al., 2016  Katsakou, 2016  Katsakou et al., 2012  Larivière, 2015  Shepherd et al., 2017  McSherry et al., 2012  Clarke, 2017  Clarke & Waring, 2018  Gillard et al., 2015 |
| **Theme 7:** Group therapy: A setback  **This theme reflected a preference among a sub-group of participants to withdraw and distance themself from the group component of their treatment and group therapy. This was because group therapeutic settings that embrace belonging and friendship-making were perceived as threatening, rejecting or/and alienating, or even a setback in terms of recovery.** | 1.Recovery and discovery – doing things differently:  Interviewees also recognised that setbacks in  recovery could result from feeling judged, rejected or not understood in their relationships  2.Managing complex group processes:  - Her (participant) accounts providing a sense of this (group therapy) as lacking or inconsistent  3.Confronting interpersonal difficulties and practising new ways of relating:  - Other common experiences included being disheartened after listening to other people’s difficulties and seeing oneself as belonging to a group of people with problems  4. Fear and longing:  -This sense of flow, however, was described as disappearing at the moment any thought of evaluation by others entered their minds. Thus, the moments of movement and development seemed rather fragile and fragmented.  5.Inclusivity within rituals: solidarity through negative transient emotions:  -Though physically present at the table, some clients would withdraw from the conversation, watch others eat, or pick at their food. | *“I think if you’re put in a situation where people would get to know me, I think that could have a negative effect on my recovery and not a positive one.”*  (participants stated regarding the group therapy:) *“I wouldn’t say supportive, but...”*  *“It is more like a sense of flow, and there is a feeling of progress too”* | Gillard et al., 2015  Bradley-Scott, 2017  Katsakou, 2016  Sorenson et al., 2019  Clarke & Waring, 2018 |
