# Supplementary material for: The experience of loneliness among people with a “personality disorder” diagnosis or traits: a qualitative meta-synthesis
Source: BMC Psychiatry. 2022 Feb 17;22:130. doi: 10.1186/s12888-022-03767-9 (PMC8855579; doi:10.1186/s12888-022-03767-9)
Supplement: Supplementary file 3 — Additional file 3. [file 12888_2022_3767_MOESM3_ESM.docx]

**Appendix 1: Search strategy**

**Concept 1: Personality disorder**

MeSH terms: Personality disorder (exp)

Personality disorder* or borderline personality* or emotionally unstable personality* or impulsive personality* or histrionic personality* or narcissistic personality* or antisocial personality* or dissocial personality* or paranoid personality* or schizoid personality* or schizotypal personality* or avoidant personality* or anxious personality* or dependent personality* or obsessive compulsive personality* or anankastic personality* or sociopathic personality* or mixed personality disorder* or Cluster A personality* or Cluster B personality* or Cluster C personality*or unspecified personality*

AND

**Concept 2: Loneliness**

MeSH terms: Lonely (exp)

OR lonel* or perceived social isolation or social isolat* or emotional isolat* or social network* or social support or social contact or social relation* or social capita or alienat* or social interact* or perceived social support or subjective social support or social activ* or confiding or confide or personal recover*

AND

**Concept 3: Qualitative method**

MeSH terms: qualitative method (exp)

Qualitative* or lived experience or experience or interview* or focus group* or IPA or interpretive* or grounded theory or narrative* or Discourse* or Thematic* or Content Analysis or Ethnograph* or Phenomenolog* or Hermeneutic or semistructured* or semi-structured or unstructured* or guided interview* or guided discussion* or group discussion or transcribe or open-ended or mixed method or mixed-method or framework approach
